# Reception of Dietary and Other Health-Related Lifestyle Advice to Address Non-communicable Diseases in a Primary Care Context: A Mixed-Method Study in Central Argentina

**DOI:** 10.3389/fnut.2021.622543

**Published:** 2021-01-27

**Authors:** Raúl E. Sánchez Urbano, Ariel Paredes, Frank R. Vargas Chambi, Pedro Guedes Ruela, David E. V. Olivares, Benicio T. Souza Pereira, Sandaly O. S. Pacheco, Fabio J. Pacheco

**Affiliations:** ^1^Center for Health Sciences Research, School of Medicine and Health Sciences, Universidad Adventista del Plata, Libertador San Martín, Argentina; ^2^Institute for Food Science and Nutrition, Universidad Adventista del Plata, Libertador San Martín, Argentina

**Keywords:** health-related advice, non-communicable diseases, diet, lifestyle habits, mixed- method study, primary health care, Argentina

## Abstract

An effective way to address risk factors for non-communicable chronic diseases (NCD) and reduce healthcare costs is by using sound health-related advice (HRA) to promote healthy lifestyle habits. In Argentina, however, few studies have examined the context in which HRA is communicated and undertaken by patients at the primary care level. In this study, we assessed the reception of HRA using a mixed-method approach in a central area of Argentina. A total of 1,044 participants from the community were contacted and sociodemographic characteristics, health-related lifestyle factors, and medical history were collected. A calendar with health messages was provided to participants and its usage was assessed after 1 year. Additionally, semi-structured interviews were conducted with 34 patients attending a local primary healthcare center. The results show that HRA was given more frequently to individuals with higher mean age, lower educational level, and to females. Participants with a chronic health condition are at a higher chance of receiving advice to reduce salt intake and maintain a healthy weight. Dietary advice is offered along with other lifestyle recommendations. The use of alcohol and tobacco is usually addressed together. HRA was primarily received in the context of an NCD diagnosis and advice was directed, especially, to risky behaviors. The HRA to increase the intake of fruits and vegetables was mentioned less frequently. Patients at the healthcare center greatly appreciated receiving an HRA, especially when given in a tailored, written, and detailed form, and acknowledged its importance to prevent or control a chronic health condition as part of the medical treatment but showed concern regarding the ability to fully incorporate the advice. Lifestyle recommendations are highly appreciated by patients but are still underutilized since they are offered mostly in the context of illness. The health calendar was shown to be useful to complement health intervention programs at the community level. The findings of our study underscore the acknowledged value of HRA by participants to tackle the risk factors of chronic diseases. If properly used HRA constitutes a simple and highly valued tool to help address patient's needs to prevent and control NCD in Argentina.

## Introduction

Non-communicable chronic diseases (NCD) represent an important cause of mortality around the world. According to the Ministry of Health in Argentina, NCD are responsible for ~78% of the death in the country. The most frequently diagnosed NCD in Argentina are hypertension, type 2 diabetes, and hypercholesterolemia, which are all associated with modifiable risk factors, such as an unhealthy diet, alcohol and tobacco consumption, inadequate sleep, and insufficient physical activity (1, 2).

Several studies found that an effective way to fight the risk factors associated with the development of NCD and decrease costs in healthcare is using sound health-related advice (HRA) for assisting patients with the adoption of healthy lifestyle habits (3–6). Primary healthcare centers (PHC) are in direct contact with the community and represent a strategic environment to foment healthy behaviors. The type and the way information is provided by health professionals are key aspects for supporting the patient's decision to incorporate new lifestyle habits to prevent or control NCD (7, 8).

To be effective, HRA must be clear, concise, friendly, and adapted to the individual needs considering patients' health conditions, risk factors associated with lifestyle, and sociodemographic characteristics such as the educational level, socioeconomic factors, employment status, family context, among other (9–11). Altogether, these factors may influence the patient's perception and comprehension of HRA and determine the receptivity and the understanding of the information (12, 13). The development of written and verbal communication skills by health professionals is warranted for optimal HRA conveyance (14, 15). Also, a single piece of advice may be not enough to cover all the aspects of a patient which might include multiple risk factors. For instance, Keith et al. (16) found that patients are more likely to receive advice for smoke cessation when already presenting other modifiable risk factors.

Escalating evidence underscores the recommendations associated with healthy lifestyle practices as an important part of public health strategies for the prevention, and treatment of NCD worldwide. Nevertheless, few studies in South America have examined the frequency and the form in which health-related lifestyle advice is communicated and undertaken at the primary care level. Understanding the interactions of the different individual aspects that determine the type of advice that is offered, how the advice is received, and its perceived usefulness may be investigated with a mixed-method approach. This type of study enables people's experiences to be contrasted with the numerical data, obtaining more detailed information, if working exclusively from a quantitative point of view (17–19). Thus, the objective of this research is to assess the reception of HRA in a population from a central area of Argentina and the perception of the participants about the advice received at the primary care level using a mixed methodology.

## Materials and Methods

This is a mixed-method study, using a cross-sectional analysis of the qualitative and quantitative data collected from a health-promotion research program developed in a central area of Argentina (20). This research was conducted in the city of Diamante, between the years 2014 and 2017, province of Entre Ríos located in a semi-urban zone. The total population of Diamante in the year 2010 was 19,930 inhabitants. At the beginning of the project, each participant received an oral invitation followed by a written description of the main steps of the study. Participants signed the written informed consent. All procedures associated with this project were conducted following the international ethical standards proposed by the Helsinki protocol for human research and this study was reviewed and approved by the Research and Ethics Committee of the Adventist University of River Plate School of Medicine (N° 03-01-02/2012/2-2012). This committee is affiliated to the National Register of Health Research of the Ministry of Health (registered under the #0237), Argentina.

### Quantitative Data Collection

A total of 1,044 adults, including 365 men and 679 women, were enrolled in the first part of this study. Participants were contacted in person during community, door-to-door visits, or primary health care clinic visits during the years 2014 and 2015. Participants answered the core questions of the WHO STEPwise questionnaire on NCDs risk factors for the reception of a HRA by a medical or other health worker in the last 3 years advising on tobacco cessation, reducing the intake of salt, eating five daily portions of fruits and vegetables, reducing the intake of unhealthy fats, increasing physical activity, and maintaining healthy body weight (21). The question on lifestyle advice to cut down on alcohol consumption was extracted from the full version of the AUDIT questionnaire (22). Participants also provided information on sociodemographic characteristics (age, sex, level of education, and employment statues), lifestyle factors including smoking history, alcohol risk intake (risk alcohol consumption according to the AUDIT-C instrument, a score of ≥4 points for men and ≥3 points for women) (23), habitual daily intake of 5 or more portions of fruits and vegetables, physical activity (30 min or more of moderate aerobic activity at least three or more times per week on a regular basis), and medical history including the presence of chronic diseases diagnosed by a physician (12, 20, 21).

Anthropometric data (body weight, height, and waist circumference) were measured. Body mass index was calculated from weight (kg)/height (m^2^). Central obesity was defined as ≥102 cm in men and ≥88 cm in women. Blood pressure measurements were obtained twice and averages were calculated. High blood pressure was defined as mean systolic blood pressure (SBP) ≥140 mmHg and/or mean diastolic blood pressure (DBP) ≥90 mmHg. A peripheral venous blood sample was withdrawn (~5 mL) after overnight fasting of ~12 h for lipid and other biochemical analyses.

#### Health Calendar

Each participant of the quantitative analysis received a color-printed calendar (A3 page size) containing 365 brief health messages or tips, one per day, grouped into 12 aspects of the lifestyle, presented one per month (1. physical activity; 2. sunlight exposure; 3. adequate use of water; 4. healthy diet; 5. prevention of risk factors for mental health associated with depression and anxiety; 6. adequate rest and sleep quality; 7. environmental air and breath quality; 8. prevention of dyslipidemia; 9. prevention of hypertension; 10. prevention of type 2 diabetes mellitus; 11. prevention of risky alcohol intake and smoking cessation; and 12. hopefulness and positive emotions). The calendar contained basic health messages and notes of encouragement to improve health-related lifestyle behaviors (please, see Supplementary Material). Most of the messages were based on current medical literature for community-based health promotion and the recommendations of the WHO to control or reduce NCD risk factors. After 12 months of having received the calendar, the participants were visited throughout the year 2016 to assess the self-administration of the health calendar. Each participant was asked through a short questionnaire about the usage and appreciation of the messages of the calendar and if the health information was shared spontaneously with family, friends, or co-workers.

### Qualitative Analysis

To further explore the results obtained with the quantitative approach, semi-structured individual interviews were conducted in 34 persons (women and men) from the community in a local PHC center during some months of the year 2016 and 2017. Two individuals did not provide sufficient information to include in the analysis. Interviews were conducted by one of the researchers using open-ended questions focused on the circumstances and characteristics associated with the reception, understanding, and appreciation of the HRA by patients. All interviews were recorded in audio, with the prior signed consent of each participant. For the assessment of the qualitative information, a literal transcript of each interview was made. Then a manual coding based on an inductive thematic approach was executed, to organize the data into categories and detect emerging patterns. This process was carried out in parallel by three researchers. The researchers independently conducted a line-by-line analysis of each of the transcripts, then each of the phrases/quotes was compared to establish agreements and differences, which were discussed. Finally, the most representative phrases were chosen according to the three categories considered and illustrates the observed themes and sub-themes. Quotes of participants have been translated into English and translated back to Spanish to check for accuracy.

### Statistical Analysis

Frequencies, measures of central tendency, and dispersion for quantitative variables were calculated. Chi-square and t-student tests were used for independent samples to examine the relationship between sociodemographic characteristics and the advice received. To study the probabilities of receiving certain advice according to the current health condition, a multinomial logistic regression was performed. The regression model was generated with the escalated addition of independent variables to solve the issue of collinearity between them. Individual regression analysis was performed to determine the odds ratios (OR) and its statistical significance before the variables were added to the model. The variables age, gender, and academic level were included in all models given their possible influence in all of them. Once independent variables with possible influence over-dependent variables were determined, a general model was constructed. Later, some independent variables were eliminated according to adjusted OR and level of significance, until an optimal regression equation was achieved. This process was performed assuming each HRA as the dependent variable on each model and the following independent variables: sociodemographic characteristics (age, gender, and level of education), low alcoholic risk score on AUDIT-C, smoking history, previously diagnosed health conditions (hypertension, diabetes, hypercholesterolemia, and other chronic health conditions), anthropometric parameters (BMI and blood pressure), lifestyle parameters (non-ideal fruits and vegetable consumption, practicing of physical activity less than three times per week) and biochemical parameters (total cholesterol <200 mg/dL and LDL cholesterol <130 mg/dL). Lastly, each one of the HRA was set as an independent variable as well to determine the association between having received one HRA and the reception of the others. *SPSS* Inc., (Chicago, IL, USA) version 24.0 software was used, and *p* < 0.05 were considered statistically significant.

## Results

### Sociodemographic Characteristics and Health Advice

The 1,044 participants considered in the quantitative approach had a mean age of 43 ± 15 years old, were predominantly women, with an education level of primary school, were actively working, and married as described in a previous study with this population (20). In the bivariate analysis seen in Table 1, the advice to reduce the intake of salt, consume at least five daily portions of fruits and vegetables, reduce the intake of unhealthy fat, perform physical activity and maintain a healthy weight were offered to participants with a higher mean age than those participants that reported not having received these advice. On the other hand, the advice to abstain or decrease alcohol intake was offered to younger participants compared with those subjects that have not received this advice. The advice to consume fruits and vegetables, decrease the intake of unhealthy fats, increase physical activity, maintain a healthy weight, and abstain from or decrease alcohol intake was offered more often to women. Regarding the level of education, the advice to reduce the intake of salt, unhealthy fat, or alcohol were offered more frequently to subjects with primary educational levels. Unemployed individuals received the advice to reduce salt intake at a higher frequency, while employed subjects received more often the advice to increase physical activity (Table 1).

**Table 1:** Health-related advice according sociodemographic characteristics.

**Health-related advice**	**Sex**			**Level of education**			**Employment status**	
	**Age**	**Male**	**Female**	**Primary**	**High school**	**Tertiary/university**	**Active**	Inactive^*^
	**μ (DS)**	**% (n)**	**% (n)**	**% (n)**	**% (n)**	**% (n)**	**% (n)**	**% (n)**
Quit smoking	NS	NS		NS			NS	
Yes	42.7 (±14.5)	25.3 (90)	25.6 (170)	29 (121)	24.3 (97)	20.7 (42)	26.2 (178)	24 (82)
No	43.1 (±15.5)	74.7 (266)	74.4 (495)	71 (296)	75.7 (302)	79.3 (161)	73.8 (501)	76 (260)
Decrease salt intake	**0.001**	NS		** <0.001**			**0.006**	
Yes	47.7 (±15)	46.3 (165)	52.5 (351)	61.6 (257)	45 (181)	38.2 (78)	47.3 (322)	56.4 (194)
No	38.2 (±14.1)	53.7 (191)	47.5 (318)	38.4 (160)	55 (221)	61.8 (126)	52.7 (126)	43.6 (150)
Consume 5 daily portions of fruits and vegetables	**0.001**	** <0.001**		NS			NS	
Yes	44.3 (±15.6)	54.6 (196)	66.4 (445)	64.4 (270)	59.7 (240)	63.4 (130)	61.8 (424)	63.3 (217)
No	40.8 (±14.5)	45.4 (163)	33.6 (225)	35.6 (149)	40.3 (162)	36.6 (75)	38.2 (262)	36.7 (126)
Decrease the intake of unhealthy fat	**0.001**	**0.006**		**0.001**			NS	
Yes	45.9 (±15.1)	57.7 (206)	66.3 (445)	70.2 (249)	58.6 (235)	58.5 (120)	61.5 (421)	66.9 (230)
No	37.9 (±14.2)	42.3 (151)	33.7 (226)	29.8 (125)	41.4 (166)	41.5 (85)	38.5 (263)	33.1 (114)
Increase physical activity	**0.009**	**0.008**		NS			**0.023**	
Yes	43.7 (±14.8)	61.8 (222)	70 (470)	66.6 (279)	64.8 (261)	73.2 (150)	69.5 (477)	62.5 (215)
No	41.5 (±16.1)	38.2 (137)	30 (201)	33.4 (140)	35.2 (142)	26.8 (55)	30.5 (209)	37.5 (129)
Maintain a healthy weight	**0.001**	**0.035**		NS			NS	
Yes	44.5 (±15)	56.5 (203)	63.3 (424)	64.9 (272)	56.8 (229)	61 (125)	60.3 (413)	62.2 (214)
No	40.6 (±15.3)	43.5 (156)	36.7 (246)	35.1 (147)	43.2 (174)	39 (80)	39.7 (272)	37.8 (130)
Cease or decrease the intake of alcohol	**0.007**	** <0.001**		**0.002**			NS	
Yes	36.4 (±14.6)	15.5 (44)	5.3 (20)	15.5 (34)	7.3 (20)	6.1 (10)	10.3 (51)	7.9 (13)
No	41.5 (±14.4)	84.5 (239)	94.7 (356)	84.5 (186)	92.7 (254)	93.9 (153)	89.7 (444)	92.1 (151)

* *inactive: students, housewife, unemployed, or retired. The bold values highlights the statistically significant values of p*.

### Analysis of Health Advice, Sociodemographic, and Health Conditions

The multivariable logistic regression analysis (Table 2) showed that subjects of higher age were less likely to receive the advice to quit smoking (OR = 0.9, 95% CI = 0.8–0.9) or to stop alcohol drinking (OR = 0.9, 95% CI = 0.8–0.9), as opposed with the advice to reduce the intake of salt (OR = 1.04 95% CI = 1.02–1.06) and unhealthy fats (OR = 1.03, 95% CI = 1.01–1.04), which have a higher chance to be received as the age increases. Women were 3 times more likely to receive the advice to stop drinking alcohol (OR = 3.5, 95% CI = 1.9–6.6), while subjects with a primary educational level were 2 times more likely to receive the advice to decrease the intake of unhealthy fats (OR = 2.4, 95% CI = 1.2–4.7), and to decrease or cease the intake of alcohol (OR = 2.4, 95% CI = 1.0–5.3).

**Table 2:** Multivariable logistic regression results of receipt of health-related advice.

**Variables**	**Decrease salt intake^**a**^**	**Consume 5 daily portions of fruits and vegetables^**a**^**	**Decrease the intake of unhealthy fat^**a**^**	**Increase physical activity^**a**^**	**Maintain a healthy weight^**a**^**	**Quit smoking^**a**^**	**Cease or decrease alcohol Intake^**a**^**
	**OR (95% CI)**	**OR (95% CI)**	**OR (95% CI)**	**OR (95% CI)**	**OR (95% CI)**	**OR (95% CI)**	**OR (95% CI)**
Age	**1.04 (1.02–1.06)**	0.9 (0.9–1.0)	**1.03 (1.01–1.04)**	0.9 (0.9–1.0)	0.9 (0.97–1.0)	**0.9 (0.8–0.9)**	**0.9 (0.8–0.9)**
Female	1.3 (0.7–2.3)	0.7 (0.5–1.0)	0.9 (0.7–1.5)	0.8 (0.5–1.3)	0.8 (0.39–1.5)	0.8 (0.3–1.9)	**3.5 (1.9–6.6)**
Primary academic level	1.4 (0.7–3.1)	0.8 (0.5–1.2)	**2.4 (1.2–4.7)**	**0.5 (0.3–0.9)**	0.6 (0.24–1.5)	1.1 (0.4–3.4)	**2.4 (1.0–5.3)**
High school academic level	1.3 (0.7–2.6)	0.9 (0.6–1.4)	1.5 (0.8–2.7)	0.7 (0.4–1.2)	0.4 (0.2–1.0)	1.1 (0.4–2.9)	0.9 (0.4–2.0)
Smoking history	–	–	–	–	–	**9.0 (3.9–20.9)**	–
Low alcohol risk	–	–	–	**1.7 (1.0–2.7)**	–	–	**0.4 (0.2–0.8)**
Non–ideal fruit consumption	–	**0.5 (0.3–0.8)**	–	–	–	–	–
Physical activity < than 3 times/week	–	–	–	**1.7 (1.0–2.7)**	–	–	**3.8 (1.6–8.8)**
Previous diagnosis of HBP	**2.2 (1.2–4.1)**	–	–	–	–	–	–
Previous diagnosis of DBT	1.9 (0.9–3.9)	–	–	–	**2.8 (1.2–6.4)**	–	–
Prevous diagnosis of HCL	**2.1 (1.2–3.7)**	–	–	–	–	–	–
Other chronic health conditions	–	–	–	–	**2.7 (1.0–7.2)**	–	–
BMI <25 kg/m^2^	–	–	**0.4 (0.2–0.7)**	–	**0.05 (0.01–0.2)**	–	–
BMI >25 y <30 kg/m^2^	–	–	**0.5 (0.3–0.7)**	–	**0.3 (0.1–1.1)**	–	–
Normal blood pressure value	**0.3(0.1–0.5)**	–	–		–	–	–
Total choresterol <200 mg/dl	–	–	–	–	**4.2 (1.5–11.3)**	–	–
LDL <130 mg/dl	–	–	**0.4 (0.2–0.7)**	–	**0.6 (0.1–0.7)**	**0.3 (0.1–0.7)**	–
Decrease alcohol intake advice	–	–	–	–	–	**5.7 (1.5–21.2)**	–
Quit smoking advice	**2.6 (1.3–5.0)**	**1.7 (1.1–2.4)**	–	**2.7 (1.5–4.8)**	–	–	**4.8 (2.7–8.7)**
Decrease salt intake advice	–	**1.9 (1.3–2.7)**	–	–	**2.2 (1.1–4.4)**	**3.8 (1.5–9.8)**	–
Consume 5 daily portions of fruits and vegetables advice	–	–	**3.8 (2.4–6.1)**	**3.0 (1.9–4.8)**	1.9 (0.9–3.7)	**3.3 (0.1–0.7)**	–
Decrease the intake of unhealthy fat advice	–	**3.9 (2.7–5.7)**	–	**4.8 (2.9–8.1)**	**2.5 (1.1–5.4)**	–	–
Increase physical activity advice	**3.4 (1.6–7.2)**	**2.9 (2.0–4.1)**	**8.2 (4.7–14.2)**	–	**10.3 (4.6–22.9)**		–
Maintain a healthy weight advice	**2.1 (1.1–4.0)**		**3.2 (1.9–5.4)**	**6.1 (3.8–9.8)**	–		–

Bold font: the variable is significant with p < 0.05.

OR, Odds ratio; CI, confidence Interval; HBP, High Blood Pressure; DBT, Diabetes; HCL, High Cholesterol Level; BMI, Body Mass Index.

a *Each stratified model includes demographic characteristics (Age, Sex, Academic level), health behaviors (smoking history, fruits, and vegetables consumption, alcohol risk) health conditions (previous diagnosis of HBP, DBT, HCL), Biochemical and anthropometric parameter (BMI, blood pressure value, total cholesterol values, LDL values) and HRA interactions*.

Reducing salt intake is the advice with a greater association with a previous medical diagnosis, having 2 times more chance to be offered to subjects with a diagnosis of hypertension (OR = 2.2, 95% CI = 1.2–4.1) or elevated cholesterol (OR = 2.1, 95% CI = 1.2–3.7). Having a previous diagnosis of diabetes increases the odds of receiving the advice to maintain a healthy weight by 3 times fold (OR = 2.8, 95% CI = 1.2–6.4). Regarding the BMI, subjects with normal weight or overweight presented less chance to receive advice to reduce the intake of unhealthy fat (OR = 0.4, 95% CI = 0.2–0.7; OR = 0.5, 95% CI = 0.3–0.7, respectively) and to maintain a healthy weight (OR = 0.05, 95% CI = 0.01–0.2; OR = 0.3, 95% CI = 0.1–1.1, respectively). Presenting normal LDL cholesterol levels decrease the chance of receiving the advice to quit smoking (OR = 0.3, 95% CI = 0.1–0.7), decrease the intake of unhealthy fats (OR = 0.4, 95% CI = 0.2–0.7), and maintain a healthy weight (OR = 0.6, 95% CI = 0.1–0.7).

Advice related to dietary habits is more likely to be administered along with advice to increase physical activity and maintain a healthy weight. Regarding the use of tobacco, the advice to quit smoking was associated with advice related to diet and physical activity. On the other hand, the advice to decrease alcohol intake is usually provided along with the advice to quit smoking and vice-versa (Table 2).

### Usage of the Health Calendar

Of the 1,044 participants, 86% (*n* = 824) answered the calendar usage assessment questionnaire after 1 year or more of being first contacted. The remaining 14% (*n* = 220) could not be personally located during two or three consecutive visits, or due to an uninformed change of address or discontinuation of participation in the study. Almost 45% of participants informed having read the calendar weekly. According to the use of the calendar, 15% (*n* = 124) of all participants read it daily, 15.8% (*n* = 130) read it 2 or 3 times a week and 13.7% (*n* = 113) read it once a week. Half of the participants that read the calendar weekly used to share the health tips they considered useful with family members and friends. Of the subjects that read the calendar daily, 50% shared the health advice with family members, 17% with friends, and 9.7% with coworkers. Out of subjects that read the calendar between 2 and 3 times a week, 55.4% shared the advice with family members, 16.2% with friends, and 10% with coworkers. And out of the ones that read the calendar once a week, 38.9% shared the advice with family members, 18.6% with friends, and 10.6% with coworkers. The other half of the participants of our study read the health calendar only occasionally, every other week or monthly. Out of the 12 topics presented in the calendar, 3 of them were reported by the participants as the most relevant and were associated with healthy eating (48.7%), followed by physical activity (29.7%), and the adequate use of water (27.8%).

### Qualitative Assessment of the In-Depth Interviews

In total, thirty-two participants answered and completed the entire interview. The sociodemographic information of the interviewed participants is presented in Table 3. The analysis of the information collected during the interviews was classified according to the categories presented in Table 4 and includes: (a) Context or circumstance, which refers to the health situation presented by the participant at the time of the reception of a HRA; (b) Welcoming, which aims to know if the patient appreciated the reception of HRA given by the health professional and if such was clear and detailed enough as to be put into practice; (c) Practice, to determine whether patients had applied lifestyle changes and decision-influencing factors. Tables 5–7 shows representative quotes of the participants according to the described categories and themes.

**Table 3:** Sociodemographic characteristics of the interviewed participants.

**Characteristics**	**μ (DS)**
Age	41.9 (16.7)
Sex	**n (%)**
Female	21 (61.8)
Male	13 (38.2)
Level of education	
Primary	20 (58.8)
High school	13 (38.2)
Tertiary/University	1 (2.9)
Employment status	
Active	12 (35.3)
Inactive*	22 (64.7)

*Inactive: students, housewife, unemployed, or retired.

**Table 4:** Principal categories and themes identified in the qualitative analysis.

**Principal categories**	**Themes**
Context during the introduction to the HRA	• Health issues
	• Thematic addressed
	• Providers and delivery method
Welcoming HRA	• Patient appreciation for HRA and Professional-Patient relationship
	• Detailed Advising
Turning HRA into practice	• Difficulties on practicing HRA
	• Sharing the health advice

HRA, health-related advice.

**Table 5:** Context during the introduction to the health-related advice.

**Themes**	**Quotations**
Health issues	“*When I was diagnosed with high blood pressure, that's when they gave me all the advice. I also had elevated cholesterol, so they proposed these changes, saying that I had to start walking and all that” (62yo male)*.
	“*When I was diagnosed with different types of diseases, such as my diabetes. I also have chronic obstructive pulmonary disease, because of my smoking issues” (63yo male)*.
	“*[They gave me some advice] during my pregnancy. Before that, I had never received any health advice” (22yo female)*.
	“*I received advice when I came because of my acne, I was starting to have a lot of it” (22yo male)*.
Thematic addressed	“*Depending on the type of food, I use lemon a lot. If it's a salad or a piece of meat I give it a squeeze of lemon and it is enough to flavor it. Salt is not indispensable, it can be replaced” (50yo female)*.
	“*I started to incorporate certain things I did not consume before, for instance, fruits and vegetables” (63yo female)*.
Providers and delivery method	“*Well, the physicians told me orally, but the nutritionist gave me a folder with written detailed explanation and all that” (38yo male)*.
	“*Yes, I received written advice from the nutritionist, I've seen six or seven of them, they have a lot in common. I learned, for instance, the way to prepare the food. The last nutritionist I saw thought me how to cook healthier, how to, for instance, replace frying for oven baking. She also provided me with written instruction on what to prepare for each meal of the day” (36yo female)*.

**Table 6:** Welcoming health-related advice.

**Themes**	**Quotations**
Patients appreciation for HRA and professional-patient relationship	“*They always give me advice when I come, always. They receive me and advise me truly well. I chose this place because it provides me with the best for me” (51yo male)*. “*What I enjoy, in the first place, is when there is good personal treatment with one that is already old and feels lonely sometimes. I feel good here, everyone treats me very well, they are excellent professionals” (75yo female)*. “*I think lifestyle changes are more important than the pills because my doctor told me that if I reached a certain weight my blood pressure could stabilize. She never told me to stop taking my diabetes medication, but the staff from the biochemistry lab told me they know of people that have been taken out of them” (38yo male)*. “*The medication is important, but I don't reach the [glycemic] levels I need with it alone. If I don't do my part, I don't reach my required levels” (51yo male)*.
Detailed advising	“*Yes (when asked about details), she mentioned (her doctor) the risks one is exposed to when smoking. She also told me that physical activity might help normalize my blood pressure. My cardiologist told me all that. She said the blood flows better” (37yo female)*. “*I believe physicians need a little bit more of training on this area (when referring to lifestyle advice)” (56yo male)*. “*Walk, walk. That's all the advice they gave me” (57yo male)*. “*I would say they advised me like anyone would, like saying take care of your health or you have to quit smoking” (44yo female)*.

**Table 7:** Turning health-related advice into practice.

**Themes**	**Quotations**
Difficulties on practicing HRA	“*The doctors give you the information, all the explanation, but then it depends on oneself to make the change, the click” (45yo male)*.
	“*It was hard for me because one is poor and has no money to prepare different kinds of meals for everyone in the house” (75yo female)*.
	“*[I would say the reason why I'm so addicted to smoking is to] have grown up alone in the streets” (58yo female)*.
Sharing the health advice	“*I always share my medical advice with my son” (75yo female)*.
	“*Actually no, because you can't mind someone else's business. They might even tell you why do you care about what I do?” (65yo male)*.

#### Context During the Introduction to the HRA

We analyzed the circumstances surrounding the reception of the HRA, and key parameters were identified. The chief reason for the initial visit to the PHC centers to be seen by a physician was a perceived illness or symptom associated with NCD, while a general check-up was rarely mentioned. Most participants reported having received their first-ever HRA from a physician or a nutritionist. The visit to the nutritionists was always a referral from physicians. Most participants reported receiving verbal advice rather than written detailed instructions. The first symptoms of a NCD were often reported as the driving factor to seek medical attention. Commonly mentioned NCD were hypertension, elevated cholesterol levels, and type 2 diabetes mellitus. Other participants, usually younger ones, reported other types of health issues, such as obesity, pregnancy, and infectious diseases. The advice that participants mostly mentioned were on: (1) diet, with the reducing salt intake and decreasing the intake of unhealthy fats as the most prevalent among them, followed by the advice to increase the intake of fruits and vegetables mentioned by some individuals; (2) physical activity, which was rarely detailed, but with walking being the most recommended of them; (3) alcohol intake and (4) tobacco consumption. The advice on the last two categories was mostly about the cessation of those habits. In general, the advice most of the participants mentioned practicing were: (1) Reducing the direct use of salt while or after cooking. And (2) to increase the consumption of fruits and vegetables.

#### Welcoming HRA

Almost all of the participants reported having enjoyed and appreciated receiving an HRA from their providers. Some of them even associated the reception of an HRA during the visit with the perception of a better level of personal care and an improvement on the professional-patient relationship. Some participants mentioned that health-related lifestyle advice is as important as medication for the treatment of their NCD. The level of detailed explanation on the HRA varied. Most of the patients believe to have received more than enough information to help them with the treatment for the presented disease or to incorporate new habits into their lifestyle. On the other hand, some participants believed that not enough details were provided on what to eat while avoiding unhealthy fatty foods or on the type and amount of physical activity recommended. Participants believed physicians had no time or no interest in providing better details, and a few also believed professionals did not possess enough qualifications on the matter.

#### Turning HRA Into Practice

Participants were asked open-ended questions on their opinions about what the greatest difficulties were faced in applying the HRA. According to the majority of them, the lack of personal effort was the greatest factor, followed by economic difficulties to, for instance, purchase the appropriate healthy food or to create a balanced daily menu. Other less commonly mentioned difficulties include the lack of time due to excess of work and the socioeconomic environment of prevalent constraint and toxic habits. Many participants reported sharing the received HRA with their families and friends. Some participants considered these subjects to be sensitive enough to prevent them to share.

## Discussion

This study revealed that dietary and other health-related lifestyle advice is given more frequently to individuals with higher mean age, lower educational level, and to females. Patients with a chronic health condition are at a higher chance of receiving advice to reduce salt intake and maintain a healthy weight and, in general, dietary advice is offered along with other recommendations to maintain a healthy weight, increase physical activity, and quit smoking. The HRA to limit the consumption of alcohol is usually addressed along with the advice to abandon the use of tobacco. The qualitative analysis showed that HRA was primarily received in the context of a diagnosis of an NCD and that advice was directed, especially, to risk behaviors associated with reducing the intake of salt and unhealthy fats, and to avoid the consumption of alcohol and tobacco. Patients mentioned less frequently the reception of advice to increase the intake of fruits and vegetables. Patients greatly appreciated receiving an HRA, especially when they are given in a tailored, written and detailed form, and acknowledged the importance of HRA to prevent or control a chronic health condition as part of the medical treatment, but showed concern regarding the ability to incorporate them into their daily lives.

The differences in the prevalence of the HRA according to sociodemographic characteristics allow the identification of areas to reinforce to reduce such differences in the offering of lifestyle recommendations. In this study, between 60 and 70% of women received the advice to consume five daily portions of fruits and vegetables, decrease the intake of unhealthy fats, increase physical activity, maintain a healthy weight. These findings were similarly observed in other studies (24, 25). The reason for the gender-based difference in prevalence may be because women are more interested in healthcare-related information, even though men usually have more risk factors associated with NCD (26, 27), data corroborated in our previous study in this population (20). These results demand new strategies to incentive male subjects on having a greater interest in adopting healthy lifestyle habits.

In our study, while younger subjects were more frequently advised about the consumption of toxic substances, dietary and other HRA were directed to older participants and those with a chronic health condition, who had 2–3 times greater odds of receiving such advice. The younger healthy people are usually not aware of the consequences of unhealthy dietary choices, lack of physical activity, inadequate rest, and the use of toxic substances (20, 28, 29). Thus, for prevention purposes, there is a need to promote healthy habits associated with the diet and with other aspects of the lifestyle from the early stages of life, focusing on the health education of children and adolescents (30). Similar results were found by Zwald et al. (25), where only 30% of the younger subjects received advice to increase physical activity in contrast with 60% to 70% of older subjects, with even greater chances of receiving a health advice if the person presented a NCD.

Other studies have reported that subjects with a secondary education degree or higher are more likely to receive advice to increase physical activity and to reduce the intake of diets rich in unhealthy fats (31). However, in our investigation, we found that a lower educational level was associated with a 2 times greater chance of receiving the advice to decrease the intake of unhealthy fats and the advice to decrease or cease alcohol intake. It was shown that subjects with a low educational level presented a higher prevalence of NCD and its corresponding risk factors when compared with those at a higher academic level (32). It is relevant to note the majority of the subjects in our study presented a low educational level and, hence, they may be more vulnerable to risk behaviors associated with chronic diseases. We also observed that subjects currently employed were more likely to receive advice to increase physical activity than those unemployed, retired, or in an unpaid activity. The reception of such advice may be related to specific types of jobs where sedentary behaviors are common (33, 34).

Presenting a previous diagnosis of a NCD was a significant determinant for the reception of HRA. The qualitative data of this study indicated that most patients received their first professional-provided HRA at the time a chronic disease was also diagnosed. This corroborates the findings from the quantitative analysis and points to the necessity to create primary prevention interventions designed to include patients without a previous diagnosis of NCD especially those with several risk factors and behaviors (35). Recent evidence is linking unhealthy dietary and other-lifestyle habits with low chronic grade inflammation (36), which ultimately may lead to the development of NCD. Several strategies may be used in the PHC context to identify these risk behaviors, but a simple and effective approach may include the assessment of unhealthy habits for all patients by using validated widespread questionnaires such as the STEPwise of the WHO. This last is the WHO-recommended framework approach for NCD surveillance to get started in NCD prevention and control activities. It builds a common strategy for defining core variables for surveys, surveillance, and monitoring instruments. The objective is to achieve data comparability over time and between countries. It is a simplified approach providing standardized materials and methods as part of technical collaboration with countries, especially those that lack resources (21). The lifestyle advices to improve health behaviors found in the STEPwise approach concerns to the most basic, and widely reported protective factors for NCDs such as consuming 5 daily portions of fruits and vegetables (37, 38), practicing physical activity (39, 40), reducing the intake of salt (41, 42), decreasing the intake of unhealthy fats (43), maintaining a healthy body weight (44) and reducing alcohol consumption to a low/moderate amount (45), and tobacco smoking cessation (46).

To further explore the results of the quantitative analysis, a qualitative approach was used with a second independent group of persons from the same community at the PHC center. As reported by the participants of our study, the PHC physician was the health professional to offer the first advice regarding lifestyle recommendations to prevent or control risk factors for chronic health conditions. Verbal communication was the preferred way physicians used to inform a HRA. However, another health professional that assumed an important role in the communication of HRA in our study was the nutritionist and dietitians, though access to the services of such professionals may be limited in some places of Argentina (15, 47).

It is well-known and it would be expected that health professionals often provided their patients with the advice to consume, at least, 5 daily portions of fruits and vegetables (38). However, only a few interviewed patients at the PHC center mentioned the reception of this advice. Indeed, it is of note that none of the participants reported anything associated with the concept of consuming 5 portions of fruits and vegetables every day, which has been fomented by the Health Ministry of Argentina in recent years (48). In contrast, the advice to reduce the intake of salt and unhealthy fat is focused on the abandoning of a habit to avoid the development of a disease (49, 50). Consuming 5 daily portions of fruits and vegetables, on the other hand, may refer to the development of a healthy new habit. Our previous findings studying this population in Argentina indicated that only near 5% of this population had a regular intake of 5 daily portions of fruits and vegetables (20), which more recently was also observed at a larger scale through the National Health Survey of 2018 in Argentina (1, 51). These challenging data are sometimes difficult to address. A study in the UK reported that over 75% of primary care professionals acknowledged having had insufficient training to provide their patients with specific advice on nutrition and physical activity (47, 52).

According to our study, advice related to dietary habits had a higher chance to be offered along with the advice to increase physical activity and to maintain a healthy weight. Other research reported that physician advice at the primary care level is an important determinant for the practice of physical activity (52–54), which suggests the efficiency of HRA to generate lifestyle modifications and health improvements. It is of note that the current dietary guidelines of Argentina include the advice to incorporate physical activity, along with the recommended food plate, on a daily basis and also the advice to decrease the intake of salt, which average of consumption is around 11.2 grams per day in this country (55, 56).

The advice to decrease the intake of unhealthy fats and the advice to maintain a healthy weight were less likely to be offered to subjects with a BMI <30 kg/m^2^. A study from the USA reported that only between 20 and 40% of overweight subjects, free of other chronic health conditions, received any kind of HRA (29). This represents a missed opportunity to provide health advice to patients with normal weight (5), and, which is even worse, to overweight patients, showing an unnoticed risk factor that should prompt attention. Recent information shows that there is a high prevalence of overweight and obese individuals in Argentina (57). According to our previous findings, almost 69% of this population was above the normal weight, with a 35.2% prevalence of obesity (20).

Regarding the use of a calendar with health messages as a strategy to increase health literacy in the community, almost half of all participants of our study reported to be interested in reading the health tips weekly. The information was usually shared with family and, less frequently, with friends or coworkers. Interestingly, around 50% of those who regularly read the calendar were prone to share their information. A study found that attractive health messages used to be shared with friends or relatives (58). In contrast with this, the other half of the participants of our study from the community read the health calendar only occasionally. This may suggest a lack of interest in health subjects by a significant part of this population where risk factors for NCD are highly prevalent (20). Fomenting health literacy with the use of different strategies has proven to be useful to reduce health disparities in the community (59, 60) since the information not only remains on an individual level, but it is shared throughout the social environment. The use of different mediums to share health information, such as newspapers, magazines, or using e-health tools has been positively associated with the adoption of healthy lifestyle habits (11). The choice of delivering health information using a printed-material was made based on the assumption that this would reach a larger part of this population instead of using e-health tools. In the previous study of this population, only about 55% of the participants were able to interact with the investigators via Web (20). It was also interesting to see that the more rated topics pointed by participants were associated with advice on the field of diet, physical activity, and the use of water. Coincidently, the advice on similar areas was repeatedly mentioned by patients interviewed at the PHC center, although they had not previously participated in the quantitative part of this study.

The qualitative analysis indicated that patients considered most of the HRA relevant to their current medical condition, acknowledging the role such advice has on the improvement of their health. Some of them even declared that practicing the suggested lifestyle changes was more effective than taking a prescribed drug, which is a relevant fact that has already been mentioned in weight reduction studies (10). It is also been suggested that the information considered important by the health professionals will be the one to generate more interest in the community (59). The satisfaction with the detailing on the information given varied among participants, with some suggesting a perceived lack of training from the professionals on the subjects. It is important to mention that perceived satisfaction may depend on the individual educational level (61) and that even non-detailed lifestyle advice has been shown to have a certain level of effectiveness (62).

Health information alone is not enough to induce lifestyle changes (63, 64), but according to the participants of our study, the perceived quality of the relationship between health professionals and patients appeared to have an impact on the acceptance and practice of the HRA. Reported barriers to practicing the received HRA included the lack of personal effort, financial restrictions, lack of time to prepare meals or to exercise, and the lack of support from the social environment (family, friends, or neighbors). Nevertheless, participants were able to recognize most of these could be overcome with some effort on their part. Physicians providing HRA should recognize the patient's specific difficulties and attempt to tailor every advice to the patient's needs and help them to create a concrete plan to achieve new habits and promote lifestyle changes.

The limitations of this study include the evaluation of only those advice included in the STEPwise questionnaire used to quantitatively access the HRA reception in this community, and it does not take into account other types of useful advice in the prevention of NCDs such as the reduction in the intake of ultra-processed foods and processed meats, sugary foods, soft drinks, adequate intake of water, proper rest, stress management, etc. Besides, the STEPwise questionnaire asks for information regarding the last 3 years of the participants' lives, which they have to remember to answer, leading to memory-dependent bias. Regarding the calendar, we reached out to the participants after a year or more to assess the usage of the health calendar, and thus a part of them was not found. Also, this study was performed in a restricted area of Argentina, hence the results cannot be generalized to the entire population, even though the data on the prevalence of NCDs show similarities between our participants and the general population. It is valid to note that our study only considered the patient perspective on the reception of HRA, leaving aside the opinion of the healthcare professionals what may be explored in a future investigation.

The strengths of this study included the use of a mixed-method approach to assess HRA in a primary health care context of Argentina with a previously detected high prevalence of risk factors for NCD. The qualitative approach sheds light on the patient's perceptions of well-recognized health advice prescribed by medical doctors and nutritionists and expands the understanding of how HRA is seen by patients, making decisions more operative in the context of PHC. The obtained data may be strategically leveraged for prevention at all levels of health attention, including prevention, treatment, and control of chronic health conditions. The study addresses the topic of dietary and other lifestyle-related factors influencing NCD in a population of South America, a topic that needs greater attention in low-and-middle-income countries, as highlighted by a 2015 research which showed that only 0.5% of all health interventions targeting multiple risk factors for NCD were conducted in South America (65). This research highlights the importance that patients place on receiving health counseling, recognizing lifestyle changes as a key part of the prevention and treatment of NCD, and indicates the need for the training of the interdisciplinary team to fulfill with excellence the role of primary health care.

In the population of this central area of Argentina, patients previously diagnosed with chronic health conditions were more likely than others to report receiving advice to adopt healthy lifestyle recommendations. While HRA for those with hypertension, elevated cholesterol, or diabetes is an important public health goal, the results of this investigation suggest that health professionals may be missing the opportunity to engage in broader primary prevention strategies. The data also showed that a large proportion of the population, who could benefit from these recommendations, are not being advised to adopt them. It is estimated that, for instance, 65% of cardiovascular events could be prevented by the adoption of lifestyle recommendations (66) as also indicates the recently released medical consensus for the primary prevention of cardiovascular diseases (67). Thus, the information reported in this investigation may be helpful for healthcare professionals and providers in Argentina and across the world, especially in those countries with similar socioeconomic and cultural characteristics, since, according to the qualitative data, patients consider their physician's advice important, appreciate receiving them, and are prone to share the information with others. The findings of our study may be used to foster tailored-prevention strategies at the public health level using HRA to address NCD risk factors in Argentina and neighboring countries of Latin America and other areas of the world with similar challenges. Tackling lifestyle-related risk factors may provide an important boost for the economic development and if properly used HRA may constitute a simple and highly valued tool to help address patient's needs to prevent and control NCD.

## Data Availability Statement

The raw data supporting the conclusions of this article will be made available by the authors, without undue reservation.

## Ethics Statement

The studies involving human participants were reviewed and approved by Research and Ethics Committee of the Adventist University of River Plate School of Medicine (N° 03-01-02/2012/2-2012). This committee is affiliated to the National Register of Health Research of the Ministry of Health (registered under the #0237), Argentina. The patients/participants provided their written informed consent to participate in this study.

## Author Contributions

FP and SP were responsible for the design of the study. RS, AP, FV, FP, and DO participated in data collection. RS, AP, FV, FP, PG, DO, and SP participated in data analyses. BS participated in the design of the health calendar and data analyses. RS, FV, FP, PG, and SP participated in manuscript preparation. All authors contributed to the article and approved the submitted version.

## Conflict of Interest

The authors declare that the research was conducted in the absence of any commercial or financial relationships that could be construed as a potential conflict of interest.

## Abbreviations

NCD: Non-communicable Chronic Diseases
HRA: Health-Related Advice
PHC: Primary Healthcare Center
SBP: Systolic Blood Pressure
DBP: Diastolic Blood Pressure.
