# Supplementary material for: Reception of Dietary and Other Health-Related Lifestyle Advice to Address Non-communicable Diseases in a Primary Care Context: A Mixed-Method Study in Central Argentina
Source: Front Nutr. 2021 Jan 27;8:622543. doi: 10.3389/fnut.2021.622543 (PMC7873357; doi:10.3389/fnut.2021.622543)
Supplement: Supplementary file 1 [file Data_Sheet_1.PDF]

# CALENDARIO *de* SALUD

# 2015

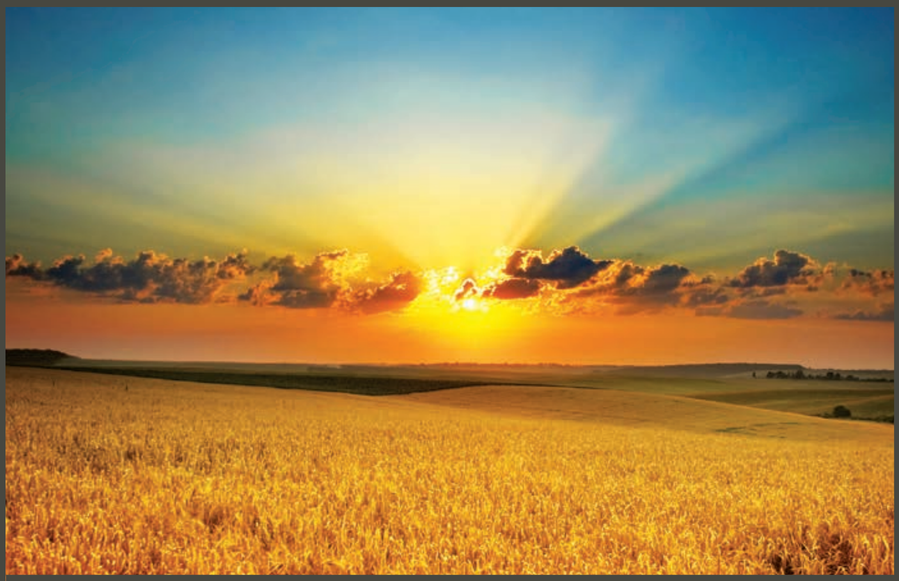
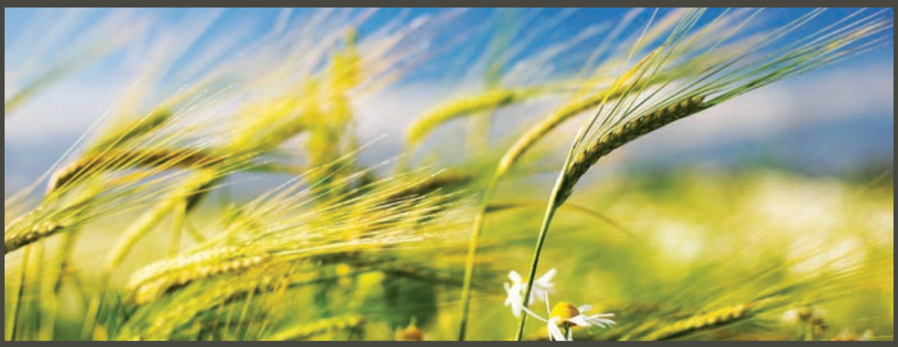
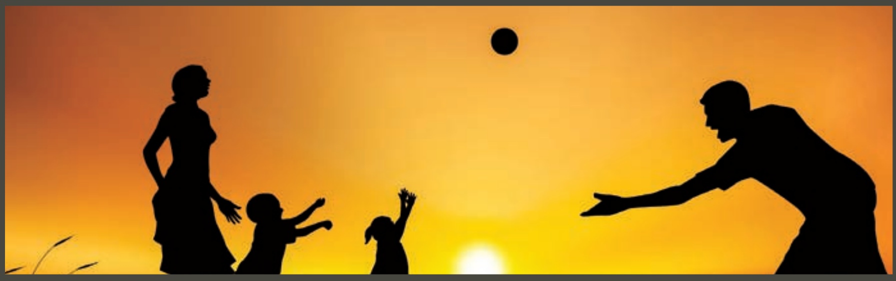
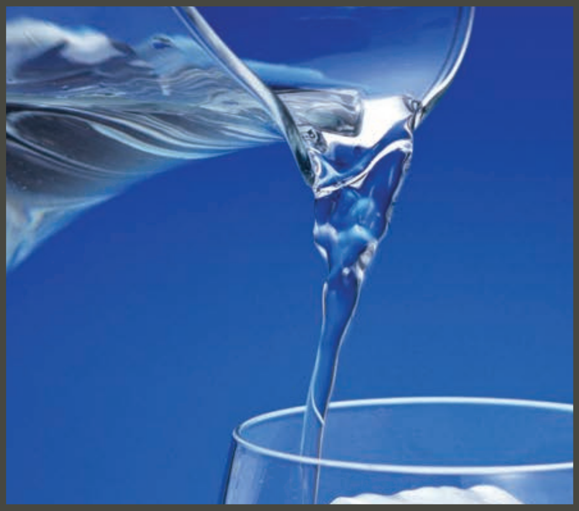
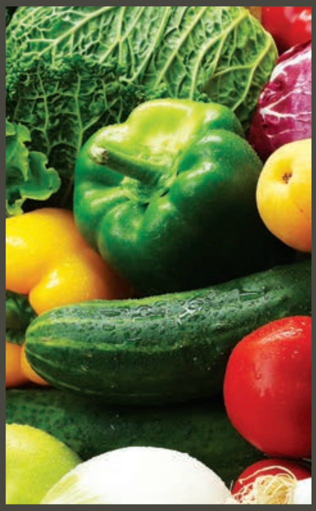
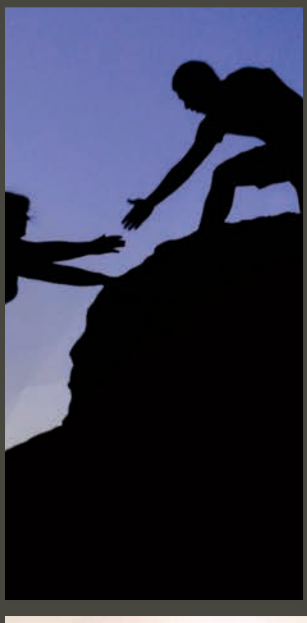
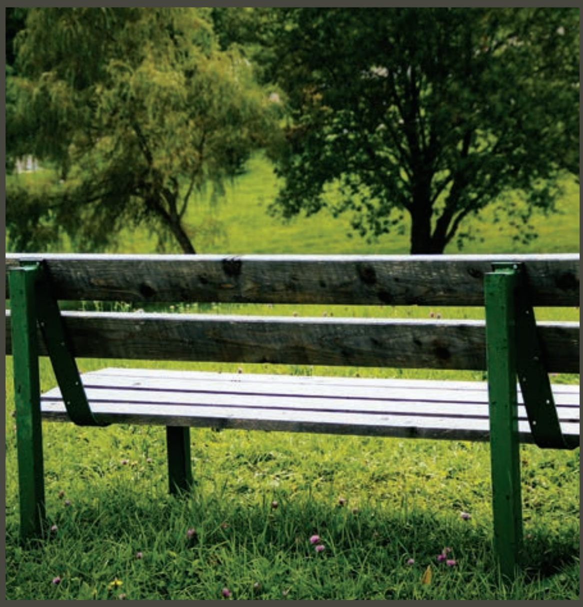
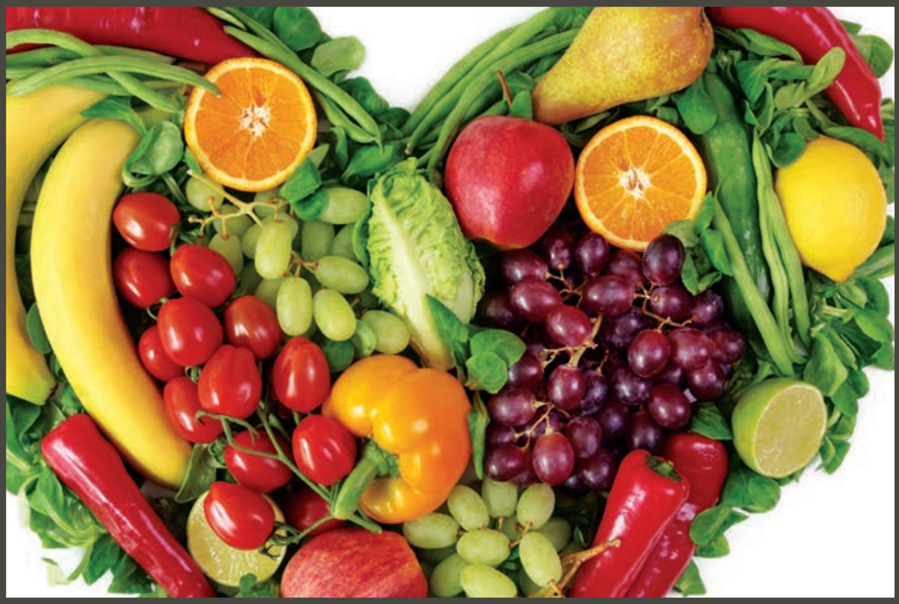
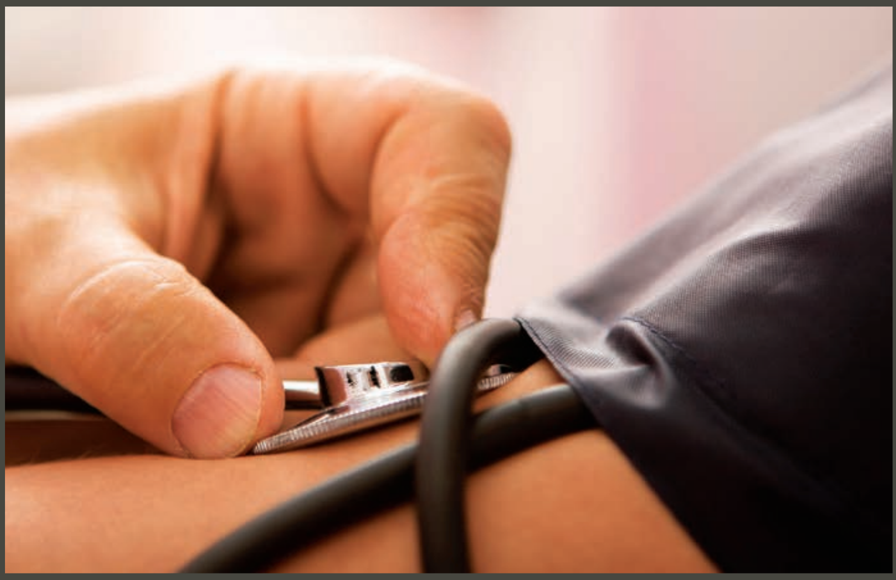
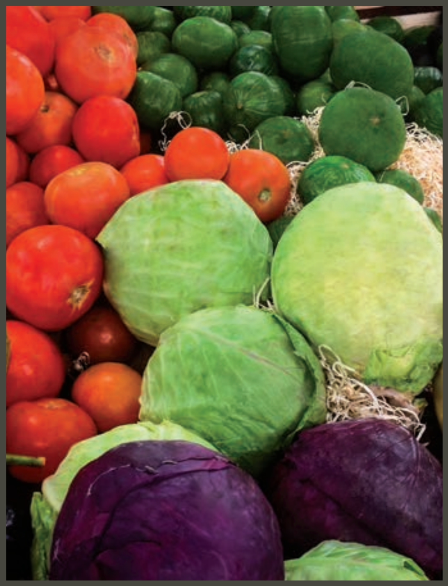
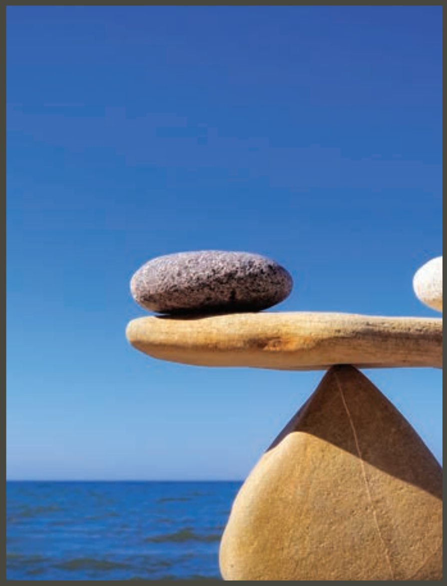
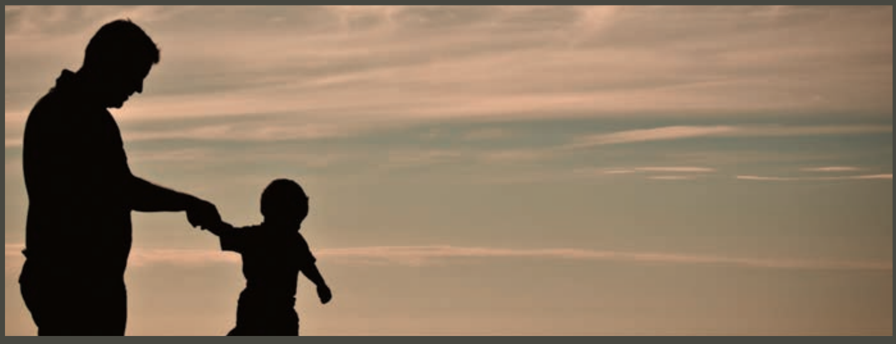

UNIVERSIDAD
ADVENTISTA DEL PLATA

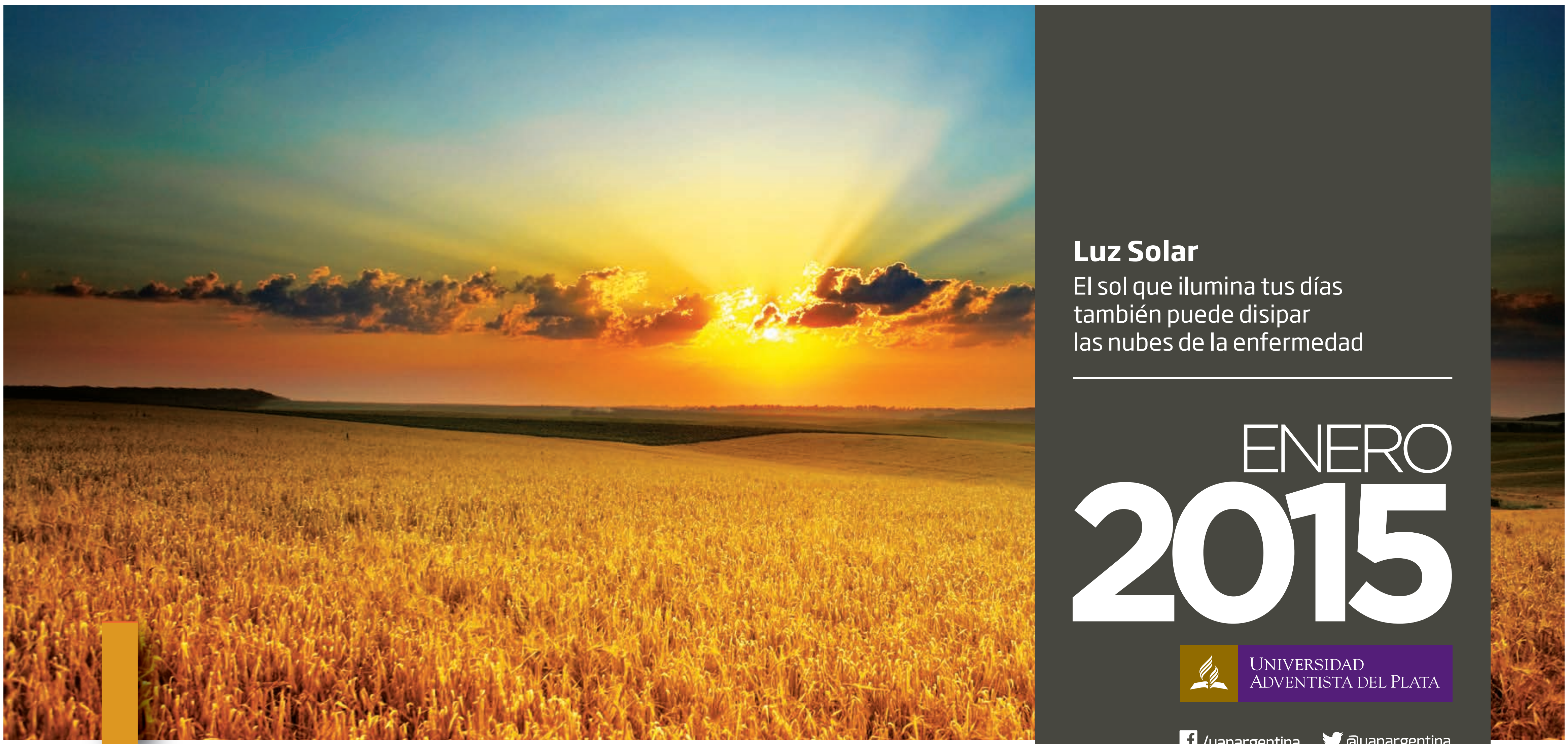

**Luz Solar**
El sol que ilumina tus días
también puede disipar
las nubes de la enfermedad

# ENERO 2015


Universidad Adventista del Plata

/uapargentina @uapargentina

25 de Mayo 99, Libertador San Martín, Entre Ríos. Argentina.
TEL: +54 343 491 8000 - www.uap.edu.ar


| DOMINGO | LUNES | MARTES | MIÉRCOLES | JUEVES | VIERNES | SÁBADO |
|---|---|---|---|---|---|---|
| Notas: | | | | **01** Asociación perfecta: sol y piel. La vitamina D es producida cuando la piel recibe sol sin interferencias, como sería detrás de un vidrio. | **02** Lo ideal es exponerse al sol son antes de las 10 de la mañana y después de las 4 de la tarde. | **03** La vitamina D favorece la absorción del calcio, fortalece tus huesos y previene la osteoporosis. |
| **04** Quince minutos de exposición al sol al aire libre, tres veces a la semana son suficientes. ¿Qué tal el sol de la mañana? | ○ **05** El exceso de sol puede causar cáncer de piel. Tómalo con moderación y evita las horas pico. | **06** Tomar sol mientras caminas por la mañana trae múltiples beneficios. | **07** No te olvides de hidratarte bien antes de estar al sol, durante y después de hacerlo. | **08** Al exponerte al sol busca tiempo y ropa adecuados. 10 a 20 minutos diarios son suficientes. | **09** La deficiencia de vitamina D duplica el riesgo de ataque cardíaco. ¡Toma sol y cuida tu corazón! | **10** Usa protector solar en cara y cuello mientras te expones al sol. |
| **11** Si tu piel es muy blanca o sensible, es recomendable consultar a tu médico antes de tomar sol. | **12** Personas de piel oscura necesitan mayor exposición al sol que las de piel clara para producir suficiente vitamina D. | ◑ **13** La fractura de cadera puede causar discapacidad y muerte entre los ancianos. Exponerse al sol ayuda a prevenir fracturas. | **14** La deficiencia de vitamina D también altera la función muscular. ¡A tomar sol! | **15** Y hay más: niveles adecuados de vitamina D disminuyen el riesgo de una tuberculosis activa y de demencia en los ancianos. | **16** Las personas expuestas regularmente al sol mantienen niveles de presión arterial más bajos. ¿Tomaste tu baño de sol hoy? | **17** Mientras más vitamina D, menor el riesgo de contraer artritis reumatoide. |
| **18** ¡Altos niveles de vitamina D ayudan a prevenir el cáncer de mama, próstata y colon! | **19** Y más: el aumento de vitamina D se correlaciona con disminución del azúcar en la sangre. | ● **20** La exposición regular a la luz del sol aumenta la amplitud respiratoria. Combina tus baños de sol con ejercicio físico. | **21** Si estás embarazada, toma algunos minutos de sol diariamente pues ayudarán a la salud de tú bebe. | **22** Estar al sol hace sudar y ayuda a eliminar impurezas por la piel. ¡Pero toma suficiente agua! | **23** ¿Sabías que exposiciones breves y repetidas son más eficientes para producir vitamina D? | **24** Otra virtud del sol es la capacidad para eliminar los gérmenes. Deja entrar libremente el sol en tu casa. |
| **25** Si hay algún enfermo en la casa, con mayor razón permite que los rayos sanadores invadan cada rincón de tu hogar. | **26** Exponerse al sol, estimula la producción de endorfinas, que producen bienestar y mejoran el ánimo. | ◐ **27** Además de hacerte sentir bien, las endorfinas mejoran tus defensas. ¡Qué agradable noticia! | **28** Las endorfinas también alivian los dolores y rejuvenecen. Buen motivo para exponerse al sol cada día, ¿verdad? | **29** El sol funciona como un tranquilizante pues favorece el tratamiento del insomnio, estrés y depresión. | **30** Televisión, computadora y video juegos te atrapan en la casa, ¿verdad? Queda al aire libre y ¡gana salud! | **31** ¿Viste como cosas tan sencillas como tomar sol cada día trae tantos beneficios? Disfruta de este remedio natural que te sale gratis. |

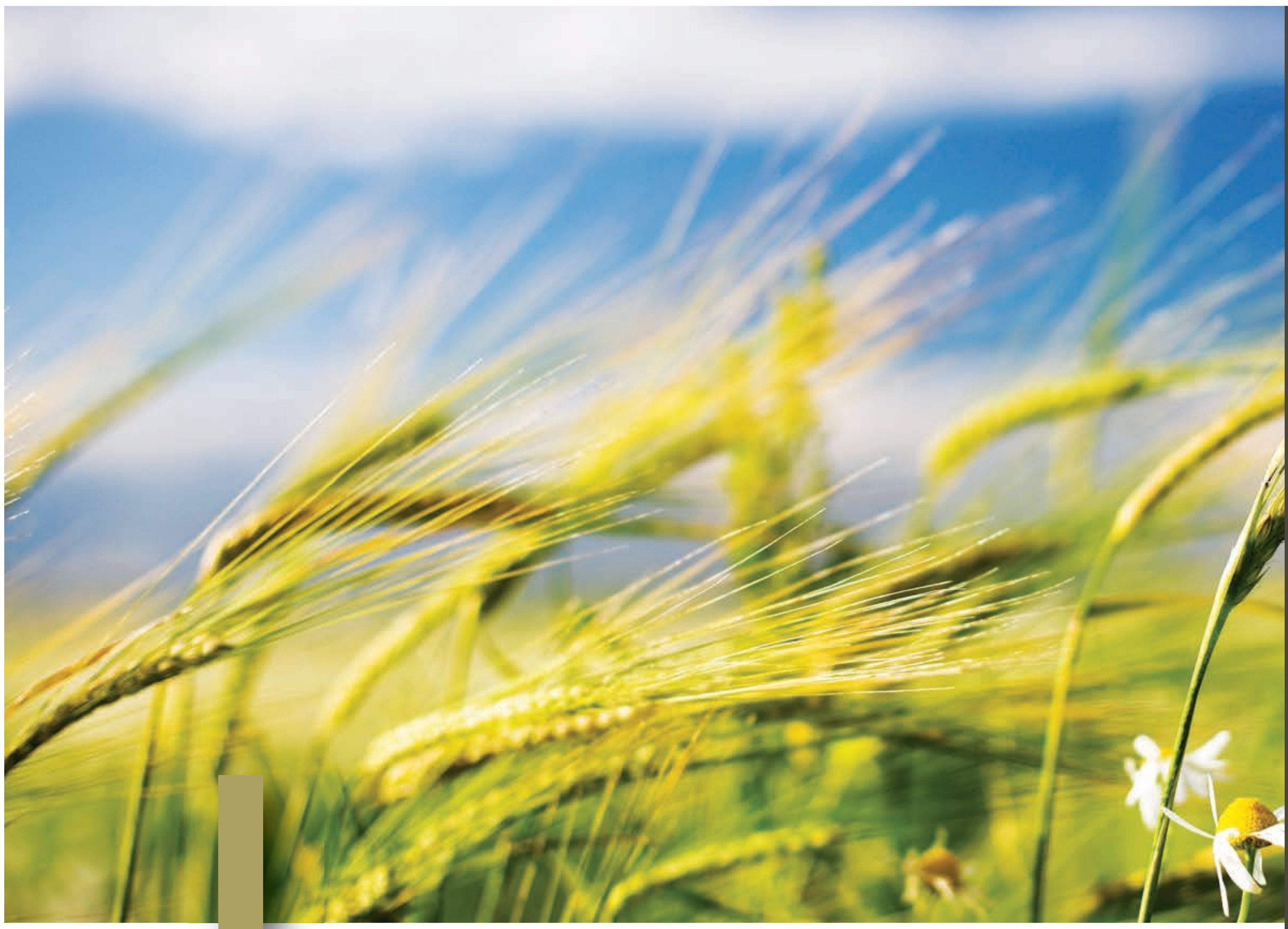

**Aire Puro y Respiración**
Necesitamos más del aire
que respiramos
que del alimento que comemos.
*(Elena G. de White)*

# FEBRERO 2015


Universidad Adventista del Plata

/uapargentina @uapargentina

25 de Mayo 99, Libertador San Martín, Entre Ríos. Argentina.
TEL: +54 343 491 8000 - www.uap.edu.ar


| DOMINGO | LUNES | MARTES | MIÉRCOLES | JUEVES | VIERNES | SÁBADO |
|---|---|---|---|---|---|---|
| **01** | **02** | **03** | **04** | **05** | **06** | **07** |
| Podemos sobrevivir varios días sin comida, unos pocos días sin agua, pero solo unos pocos minutos sin aire. | Respirar por la nariz es mejor: filtra las suciedades, calienta y humidifica el aire. | Renueva el aire de tu casa diariamente. Abre las ventanas y puertas aun en el invierno. | Respirar bien duran- te la noche es fun- damental para la salud. Busca ayuda de un médico otorrinolaringólogo. | El aire de la mañana es más puro. Temprano, haz una caminata respirando profundamente. | A causa del oxígeno respirado producimos energía. ¡Los ejercicios aeróbicos ayudan a quemar calorías! | Para oxigenar al cerebro y limpiar los pulmones inspira profundamente por la nariz y espira por la boca. |
| **08** | **09** | **10** | **11** | **12** | **13** | **14** |
| Respirar profundamente activa la circulación y contribuye a la limpieza de la sangre. | La respiración profunda ayuda a disminuir las tensiones emocionales y alivia el dolor de cabeza. | Para el enfermo es muy beneficioso permanecer algunos minutos al aire libre. | Sin doblar las rodi- llas intenta tocar tus tobillos soltando el aire por la boca. Enderézate inspirando lenta y profundamente. | El humo del ciga- rrillo daña tu salud y contamina todo el aire. Deja de fu- mar y ¡salva vidas! Inténtalo hoy. | Cultiva plantas alrededor de tu casa para purificar el aire intercambiando el gas carbónico por el oxígeno. | En el invierno, aún con calefacción prendida, deja siempre una ven- tana entreabierta para renovar el aire. |
| **15** | **16** | **17** | **18** | **19** | **20** | **21** |
| Hoy te animo a que realices 5 respiraciones profundas, inspirando lenta- mente por la nariz y espirando por la boca. ¡Tú puedes! | Mientras caminas inspira hondo contando hasta 8 y espira en el mismo tiempo. | Si sientes que no puedes respirar adecuadamente, visita a tu médico. Él te orientará hacia una solución. | Continúa tus caminatas diarias. La actividad al aire puro de la mañana mejorará tu ánimo durante todo el día. | ¿Quieres aumentar tu capacidad de concentración? Respira profundamente. | La mala calidad del aire y la respi- ración deficiente son causantes de depresión, irritabilidad, fatiga y agotamiento. | Durante el ejercicio, los pulmones intercambian 25 veces más aire que en reposo. Ejercicio y aire puro, ¡qué combinación saludable! |
| **22** | **23** | **24** | **25** | **26** | **27** | **28** |
| Mientras caminas, contempla la naturaleza y respira profundamente por un tiempo. ¡Tu día será más feliz! | La buena postura mejora la respiración. Columna recta y hombros hacia atrás aumen- tan la capacidad pulmonar. | Antes de ir a dormir, alivia tus tensiones respirando profundamente. | Con los ojos cerrados, realiza algunas inspiraciones profundas y reflexiona sobre algo importante y positivo en tu vida. | Los lugares con mucha vegetación son ideales para obtener aire puro. Busca el más cercano. | Si todavía fumas o tienes algo que entorpece el ambiente, te propongo un cambio de actitud. Tú puedes. ¡Lo lograrás! | ¡Felicitaciones! Mientras más aire, más buena disposición, más alegría y más salud. |

**Notas:**

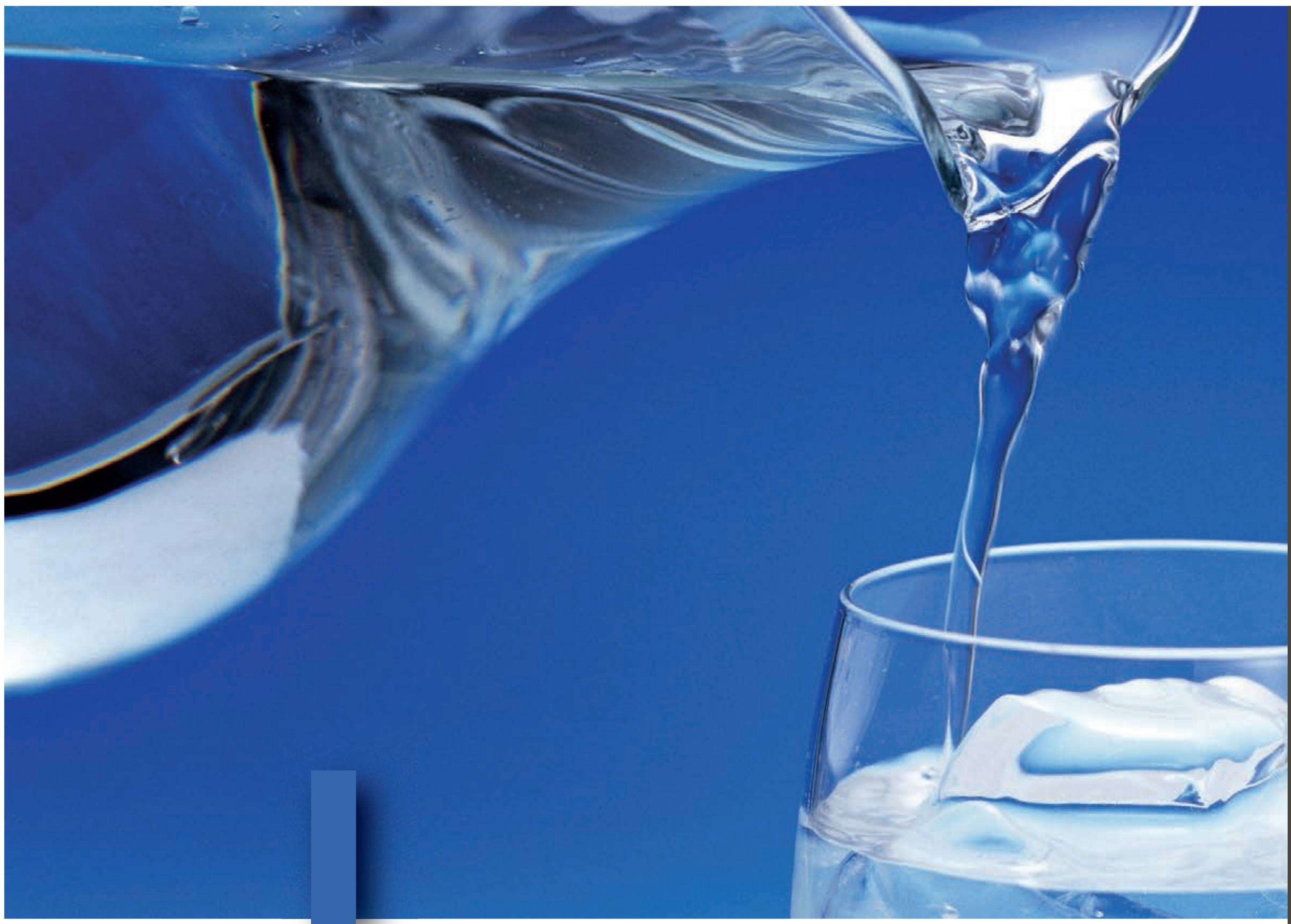

**Agua**

El agua, el aire y la limpieza son los principales productos de mi farmacia.

*(Napoleón Bonaparte)*

# MARZO 2015


UNIVERSIDAD ADVENTISTA DEL PLATA

/uapargentina @uapargentina

25 de Mayo 99, Libertador San Martín, Entre Ríos. Argentina.
TEL: +54 343 491 8000 - www.uap.edu.ar


| DOMINGO | LUNES | MARTES | MIÉRCOLES | JUEVES | VIERNES | SÁBADO |
|---|---|---|---|---|---|---|
| **01** En este mes se celebra el día mundial del Agua. Descubre la importancia del agua para tu salud. | **02** ¿Sabías que el agua es el elemento más abundante de nuestro cuerpo? | **03** Las células del cuerpo funcionan muy bien cuando están hidratadas. ¿Cuánta agua tomas por día? | **04** El agua ayuda a llevar nutrientes y oxígeno a las células. ¿Estás tomando suficiente agua? | ○ **05** Al levantarte, bebe en ayunas un vaso completo de agua fresca. Haz de esto un hábito. | **06** Los niños y ancianos se deshidratan más fácilmente. Darles agua pura los mantiene hidratados. | **07** El beber agua adecuadamente reduce el riesgo de contraer resfríos. |
| **08** El agua remueve las impurezas del cuerpo y previene el estreñimiento. | **09** Esta semana te desafío a beber al menos 6 vasos de agua al día. ¡Tú puedes! | **10** El agua provee a nuestro cuerpo gran cantidad de sales minerales. | **11** ¿Sabías que el cuerpo utiliza el agua para regular su temperatura? | **12** Beber agua adecuadamente ayuda a controlar la presión sanguínea y disminuye el riesgo de infarto. | ◑ **13** Terminar una ducha de agua caliente con un minuto de agua fría ayuda a reactivar la circulación. | **14** El 75% del cerebro está compuesto por agua. Qué importante es el agua ¿verdad? |
| **15** Casi el 85% de la sangre es agua. ¿Estás tomando tu vaso de agua en ayunas? | **16** Esta semana te desafío a consumir al menos 7 vasos de agua por día. ¡Lo lograrás! | **17** Lavarse las manos correctamente es una excelente medida de prevención de enfermedades. | **18** Beber la cantidad adecuada de agua ayuda a disminuir las complicaciones de la diabetes. | **19** El agua potable es una fuente de salud valiosa y a un precio accesible, todavía. | ● **20** Beber agua adecuadamente aumenta la capacidad mental de concentración y memoria. | **21** El 22% de la masa ósea está compuesto por agua. ¿Ya tomaste agua hoy? |
| **22** Hoy se conmemora el Día Mundial del Agua. Cuídala, ahorrando y no contaminando, por el bien de tu salud. | **23** Esta semana te recomiendo que consumas 8 o más vasos de agua por día. ¡Sé que puedes hacerlo! | **24** Cuando el cuerpo está hidratado, absorbe mejor los nutrientes de los alimentos. | **25** Consumir el agua correctamente ayuda a prevenir el cáncer de colon, mama y tracto urinario. | **26** Lavarse los ojos con agua limpia y fría ayuda a su buen funcionamiento. | ◐ **27** El agua es un gran producto de estética porque hidrata la piel desde adentro y la mantiene más tensa y sin arrugas. | **28** Además de mantener la salud, el agua ayuda a recuperarse de las enfermedades. ¡Qué medicina! |
| **29** El agua es un buen adelgazante porque ayuda a reducir los depósitos de grasa. | **30** Preferentemente bebe agua durante los intervalos entre las comidas. Es la bebida más saludable y sin contraindicaciones. | **31** Recuerda poner en práctica todos los consejos aprendidos. Brindemos con agua pura. ¡Salud! | | | | |

**Notas:**

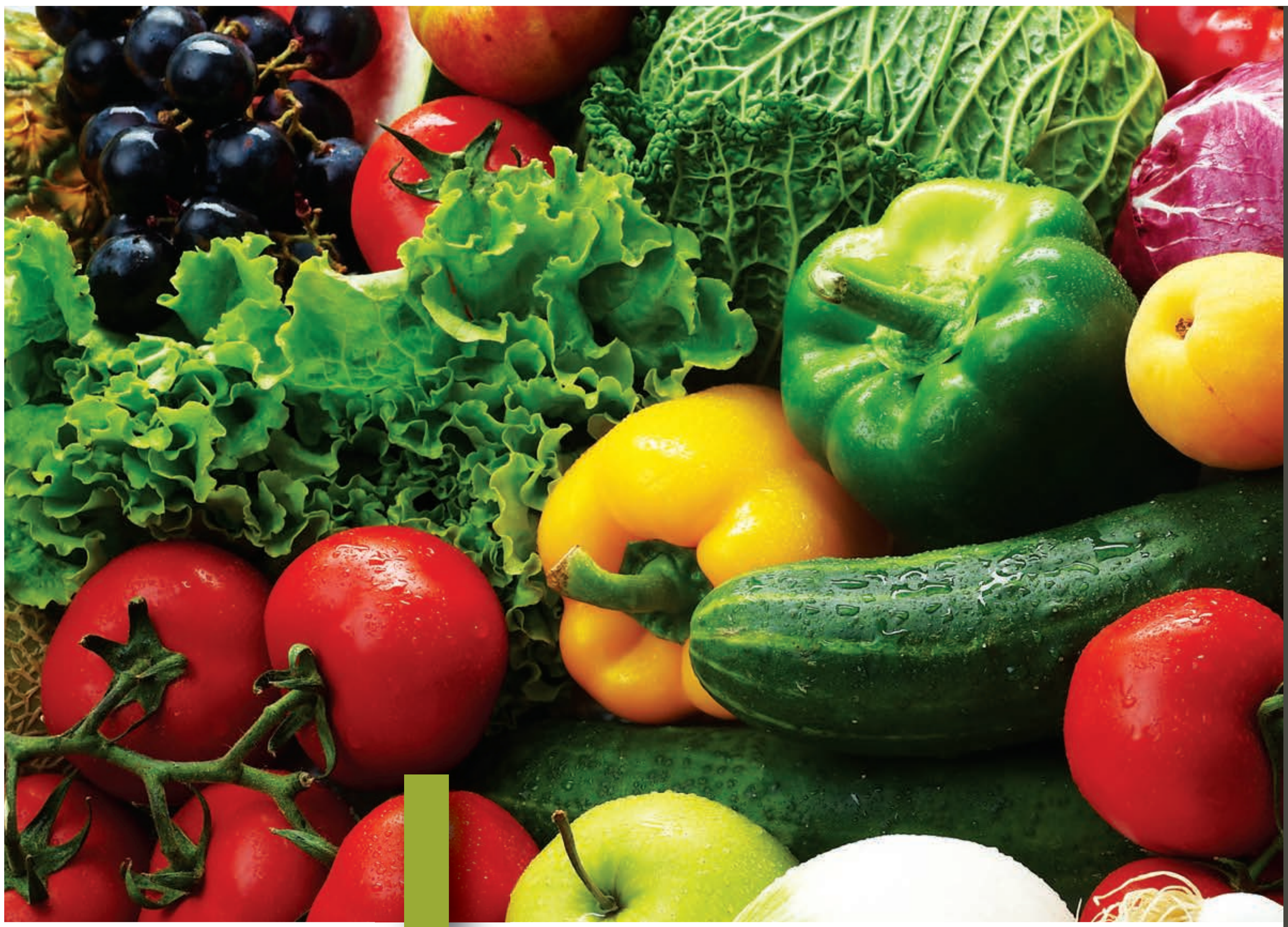

**Alimentación Saludable**
Que el alimento
sea tu medicina
y que tu medicina
sea el alimento.
*(Hipócrates)*

# ABRIL 2015


UNIVERSIDAD ADVENTISTA DEL PLATA

/uapargentina @uapargentina

25 de Mayo 99, Libertador San Martín, Entre Ríos. Argentina.
TEL: +54 343 491 8000 - www.uap.edu.ar


| DOMINGO | LUNES | MARTES | MIÉRCOLES | JUEVES | VIERNES | SÁBADO |
|---|---|---|---|---|---|---|
| Notas: | | | **01** Hay que comer para vivir, no vivir para comer. Disfruta de tu comida en un ambiente tranquilo masticando bien los alimentos. | **02** El desayuno es la comida más importante del día. Incluye frutas y cereales integrales en él para empezar bien tu jornada. | **03** Los alimentos naturales, preparados de modo simple son más saludables. | **04** ¿Consumes cereales, frutas, legumbres, verduras, hortalizas, frutos secos y semillas? ¡Un plato saludable tiene 5 colores! |
| **05** ¿Desayunaste hoy? Intenta comer en horarios regulares y no saltar comidas. | **06** La ciruela es diurética, depurativa y desintoxicante. ¡Elije una nueva fruta para probar esta semana! | **07** Conserva la salud de tus arterias. Vigila el consumo de alimentos fritos y evítalos cuanto más puedas. | **08** Las moras, entre otros frutos rojos, son ricos en antioxidantes. Consúmelos siempre para conservar tu salud. | **09** El kiwi es bueno para las encías. ¿Cómo están tus dientes y encías? | **10** Los cítricos son buenos para las arterias y ayudan a limpiar la sangre, sobretodo el pomelo. | **11** Los alimentos anaranjados previenen las enfermedades de los ojos. |
| **12** Mezclar demasiados tipos de alimentos en una misma comida propicia formación de gases. | **13** Las manzanas ayudan a combatir el mal aliento, la diarrea y el estreñimiento. | **14** El jugo de remolacha aporta minerales, reduce la anemia y el colesterol. | **15** La cebolla es excelente para el sistema respiratorio. ¿Qué te parece una rica ensalada? | **16** Las hojas verde oscuro previenen las enfermedades intestinales y la anemia. | **17** El ajo es un excelente reductor de la presión arterial y da un sabor especial a tus comidas. | **18** Las calabazas y las coles son alimentos que ayudan a prevenir el cáncer. ¿Te animas a una ensalada de brócolis? |
| **19** Para facilitar la digestión y disminuir la panza, evita el consumo líquido con las comidas. | **20** La cebada facilita la digestión y es una buena alternativa al café. Pruébala en tu desayuno. | **21** La avena equilibra los nervios y baja el colesterol. ¿Te apetecen unos copos? | **22** Por un día haz planes para una comida con vegetales, granos integrales y porotos en vez de carne. | **23** Lo ideal es combinar legumbres con cereales como arroz, maíz, mijo, o trigo. ¿Qué tal arroz con lentejas? | **24** El centeno da flexibilidad a las arterias y previene el cáncer de colon. Experimenta unas galletitas de centeno. | **25** ¡Una buena noticia para los fumadores! El germen de trigo ayuda a abstenerse del cigarrillo. |
| **26** Condimentar con hierbas y limón aumenta el sabor de las comidas y requiere menos sal. | **27** Conserva la salud de tu corazón. Reduce el consumo de margarina, manteca, embutidos y helados. | **28** El queso y la leche enteros aportan demasiado colesterol a la sangre. Cuida tus arterias. Elige los descremados. | **29** ¿Has probado la leche de soya con calcio? Es una linda y saludable alternativa a los lácteos. | **30** Tres nueces al día previenen enfermedades cardiacas y las almendras tonifican el sistema nervioso. | | |

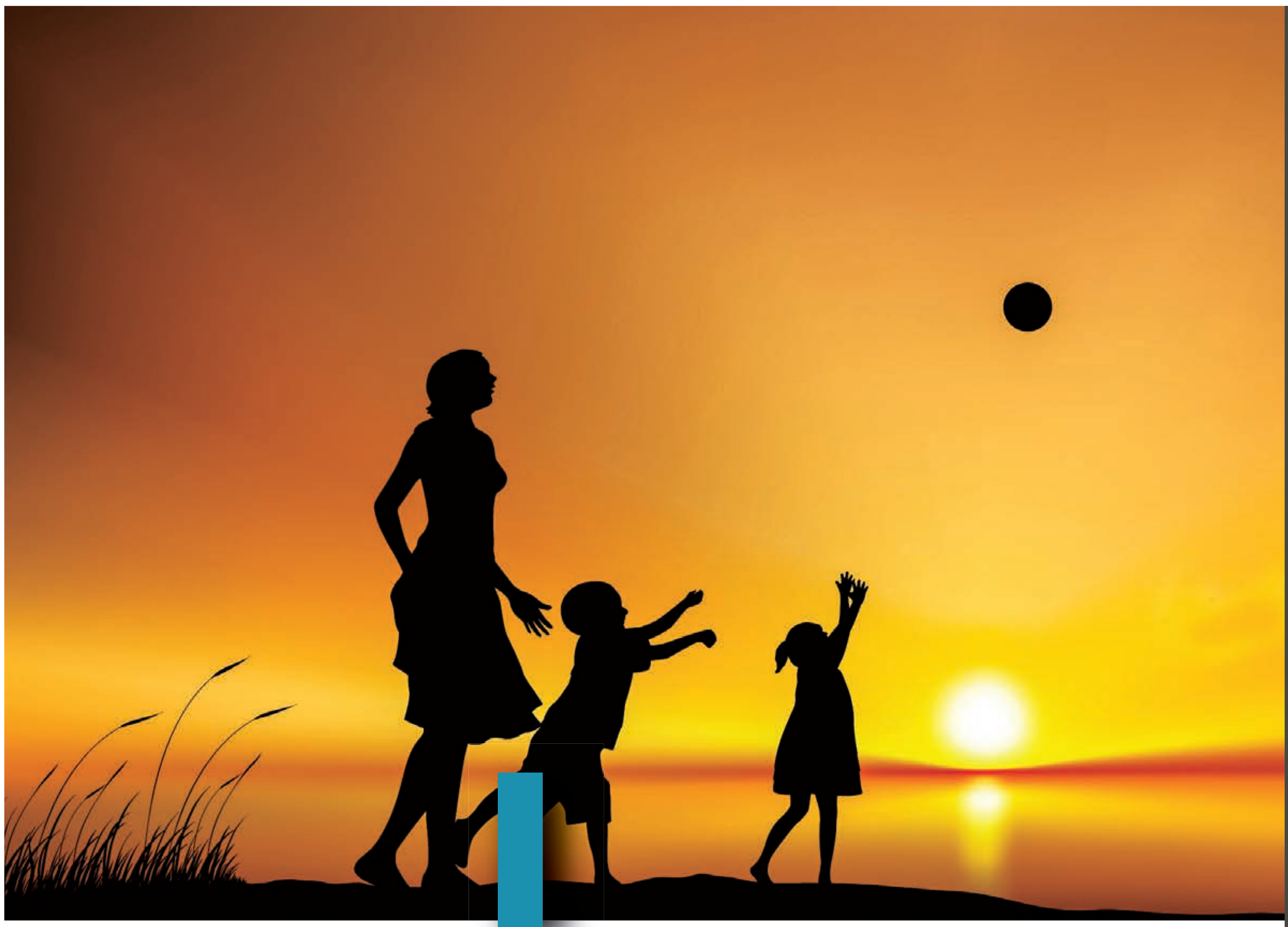

**Ejercicio Físico**
Caminar al aire libre, en horas de la mañana, es la mejor manera de prevenir centenas de enfermedades.

*(Elena G. White)*

# MAYO 2015


UNIVERSIDAD ADVENTISTA DEL PLATA

/uapargentina @uapargentina

25 de Mayo 99, Libertador San Martín, Entre Ríos. Argentina.
TEL: +54 343 491 8000 - www.uap.edu.ar


Notas:

| DOMINGO | LUNES | MARTES | MIÉRCOLES | JUEVES | VIERNES | SÁBADO |
|---|---|---|---|---|---|---|
| | | | | | **01** Viste las zapatillas, inspira hondo y activa esa preciosa máquina que es tu cuerpo. | **02** Mantente activo y ¡sorpréndete de los beneficios de la actividad física! |
| **03** La persona activa vive más y mejor. | ○ **04** El resultado de la cuenta "220 menos tu edad" es igual al máximo de latidos que puedes alcanzar durante el ejercicio. | **05** El ejercicio físico moderado disminuye la mortalidad por cualquier causa de enfermedad. | **06** Es muy importante sentirse a gusto mientras realizas actividades físicas. | **07** Caminar 20 minutos diarios es un excelente comienzo. | **08** Actividades como la jardinería, lavar el auto y barrer la casa te ayudan a quemar buena cantidad de calorías. | **09** La actividad física es benéfica aun cuando se inicia a edad avanzada. |
| **10** Si tienes problemas de salud o eres grande, consulta a un médico antes de practicar ejercicios físicos. | ◑ **11** Haz lo que más te gusta: caminata, bicicleta, natación. Lo importante es moverte. El mejor ejercicio es el que se hace con placer. | **12** Estira los músculos antes de la actividad física y después de ella. | **13** Hidrátate antes de los ejercicios y después de hacerlos. Carga una botellita con agua. | **14** Caminar fortalece el corazón. Mientras caminas, respira hondo. Hoy vas a dormir mejor. | **15** Si te duele el pecho mientras te ejercitas o si tienes sobrepeso, consulta con tu médico. | **16** Caminar al menos 1 hora por semana previene enfermedades del corazón. |
| **17** Las actividades aeróbicas son las más beneficiosas para el cuerpo. | ● **18** ¡¿Ya te estás ejercitando!? Te animo a que lo hagas con moderación para un mejor beneficio. | **19** Para sacar el mejor beneficio de la actividad física combínala con una dieta equilibrada. | **20** ¿Cómo anda tu plan de ejercicios? ¡Ánimo! Puedes empezar ahora. | **21** Te propongo estirar. Respira profundamente y "toca el cielo" con las manos contando hasta 20. | **22** Otra novedad: el ejercicio físico disminuye el riesgo de osteoporosis y de fractura de cadera. | **23** La práctica regular de ejercicio físico mejorará tu autoestima y el estrés. |
| **24/31** Recuerda: nunca es tarde para ejercitarse. La vida es movimiento. ¡Muévete! | ◐ **25** El ejercicio regular reduce el riesgo de demencia, enfermedad de Alzheimer y deterioro mental. | **26** Si elegiste la caminata, ¿qué tal si en el mismo tiempo andas una mayor distancia? | **27** Alterna momentos de la caminata con trote. ¡Los músculos y huesos van a quedar más fuertes! | **28** Te propongo otra modalidad: planifica un día de juegos en un parque o campo con la familia o amigos. | **29** ¿Sabías que mientras más ejercicio se hace menor es el deseo de fumar? | **30** ¿Problemas de estreñimiento? El ejercicio mejora el funcionamiento del intestino. |

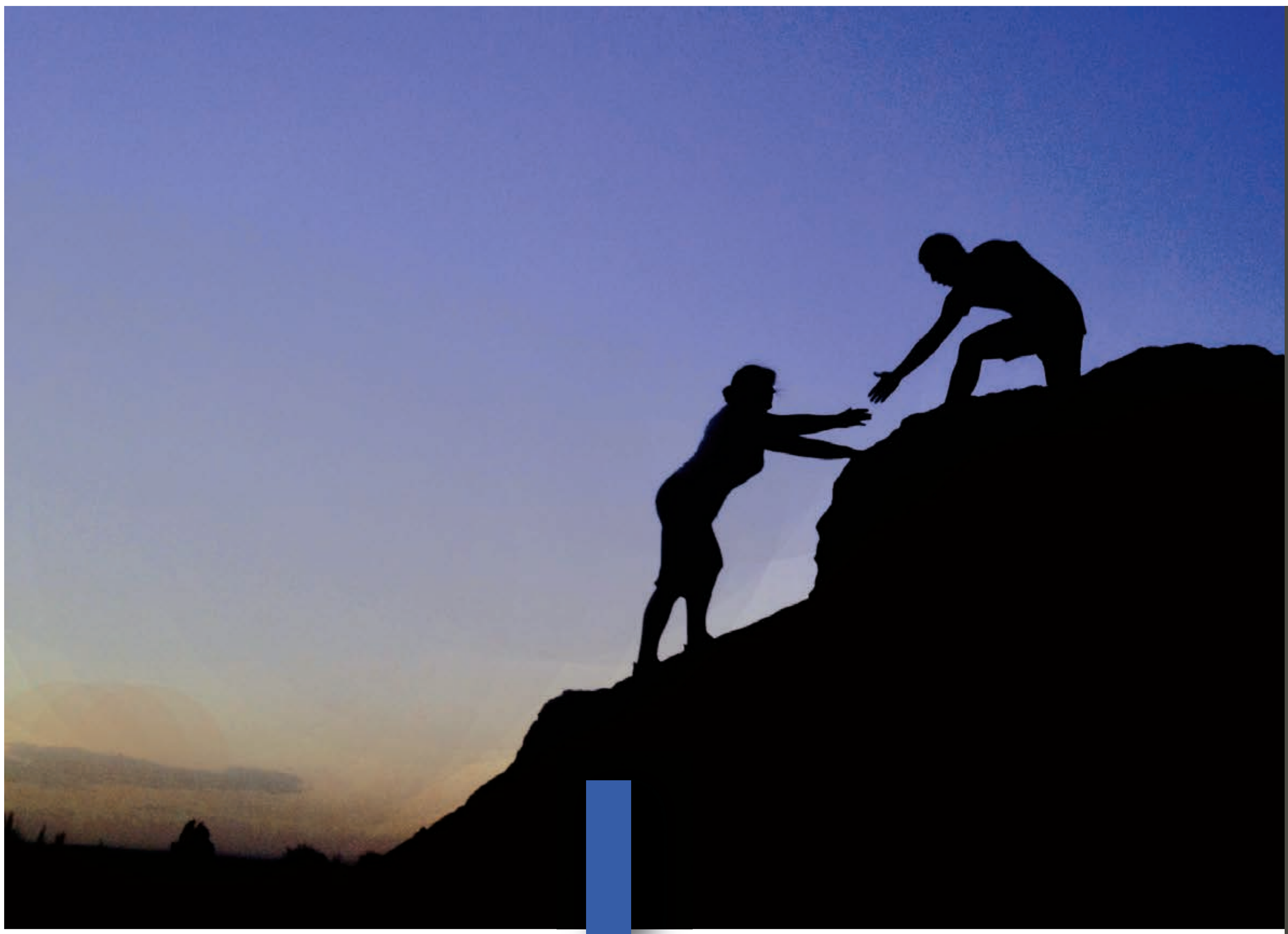

**Prevención de Depresión y Ansiedad**

Así que, no os afanéis
por el día de mañana,
porque el día de mañana
traerá su afán.
Basta a cada día su propio mal.
*(Jesucristo)*

# JUNIO 2015


Universidad Adventista del Plata

/uapargentina @uapargentina

25 de Mayo 99, Libertador San Martín, Entre Ríos. Argentina.
TEL: +54 343 491 8000 - www.uap.edu.ar


| DOMINGO | LUNES | MARTES | MIÉRCOLES | JUEVES | VIERNES | SÁBADO |
|---|---|---|---|---|---|---|
| | **01** La depresión es caracterizada por cambios en el humor y por la pérdida de placer en actividades antes placenteras. | **02** Ansiedad, como enfermedad, es la preocupación extrema por las cosas del futuro. | **03** Depresión y ansiedad son enfermedades que, tratadas eficazmente, tienen cura. | **04** La pérdida del empleo, desilusiones, divorcio y la muerte de un ser querido producen sentimientos depresivos que son normales. | **05** ¡Ojo con el estrés! Antes de que te lleve a la depresión, busca ayuda para manejarlo. | **06** ¿Te sientes triste? Prueba ayudar a alguien hoy. |
| **07** Te desafío a encontrar cosas buenas en las personas con quienes convives. | **08** Los bienes materiales no traen verdadero contentamiento. Aprende a apreciar las cosas simples de la vida. | **09** Establece objetivos nobles y dedícate a alcanzarlos. | **10** La falta de actividades placenteras es una causa de depresión. ¿Qué tal un paseo con alguien especial? | **11** Algunas medicinas causan depresión. Si presentas síntomas de depresión mientras eres medicado, habla con tu médico. | **12** El insomnio o el sueño excesivo pueden ser una señal de depresión. | **13** El tabaco, la depresión y la ansiedad están relacionados. No fumar es un factor protector contra estos males. |
| **14** Nunca te automediques por motivo de depresión. Busca un profesional de salud. | **15** La medicación ayuda a aliviar los síntomas. Para trabajar sobre la causa del problema, consulta con un profesional calificado. | **16** Conversa sobre tus sentimientos con personas con quienes tienes confianza. Asegúrate de que sean idóneas. | **17** Caminar diariamente junto a la naturaleza estimula la liberación de endorfinas contra la depresión. | **18** Toma suficiente agua pura entre las comidas para eliminar las toxinas del cuerpo. | **19** Una ducha tibia ayuda a relajarse y disminuir la tensión. | **20** Los alimentos refinados y con grasas saturadas perjudican el funcionamiento del cerebro. |
| **21** ¡Alerta! Bebidas como el café, el té negro y el mate son estimulantes del sistema nervioso. | **22** Los cereales integrales, legumbres, verduras y frutas dan energía y vitalidad al cerebro. | **23** La avena posee un precursor de la serotonina, factor importante para el funcionamiento del cerebro. | **24** Entrena tu mente a concentrarse en pensamientos positivos y a admirar lo puro, bueno, amable y digno de honor. | **25** Lo que ves, escuchas y hablas influyen sobre tus pensamientos y acciones. Nutre tu mente con cosas positivas. | **26** ¿Sabías que sonreír y abrazar estimula la liberación de endorfinas? Vive feliz y protégete de la depresión. | **27** El vivir relajado y feliz previene problemas cardíacos. |
| **28** El sentimiento de gratitud previene la depresión a través de la liberación de serotonina y endorfinas. | **29** Cultiva pensamientos de gratitud hacia las personas a tu alrededor y hacia Dios. | **30** Expresa tus sentimientos a tu Creador y ejercita la fe. | | | | |

Notas:

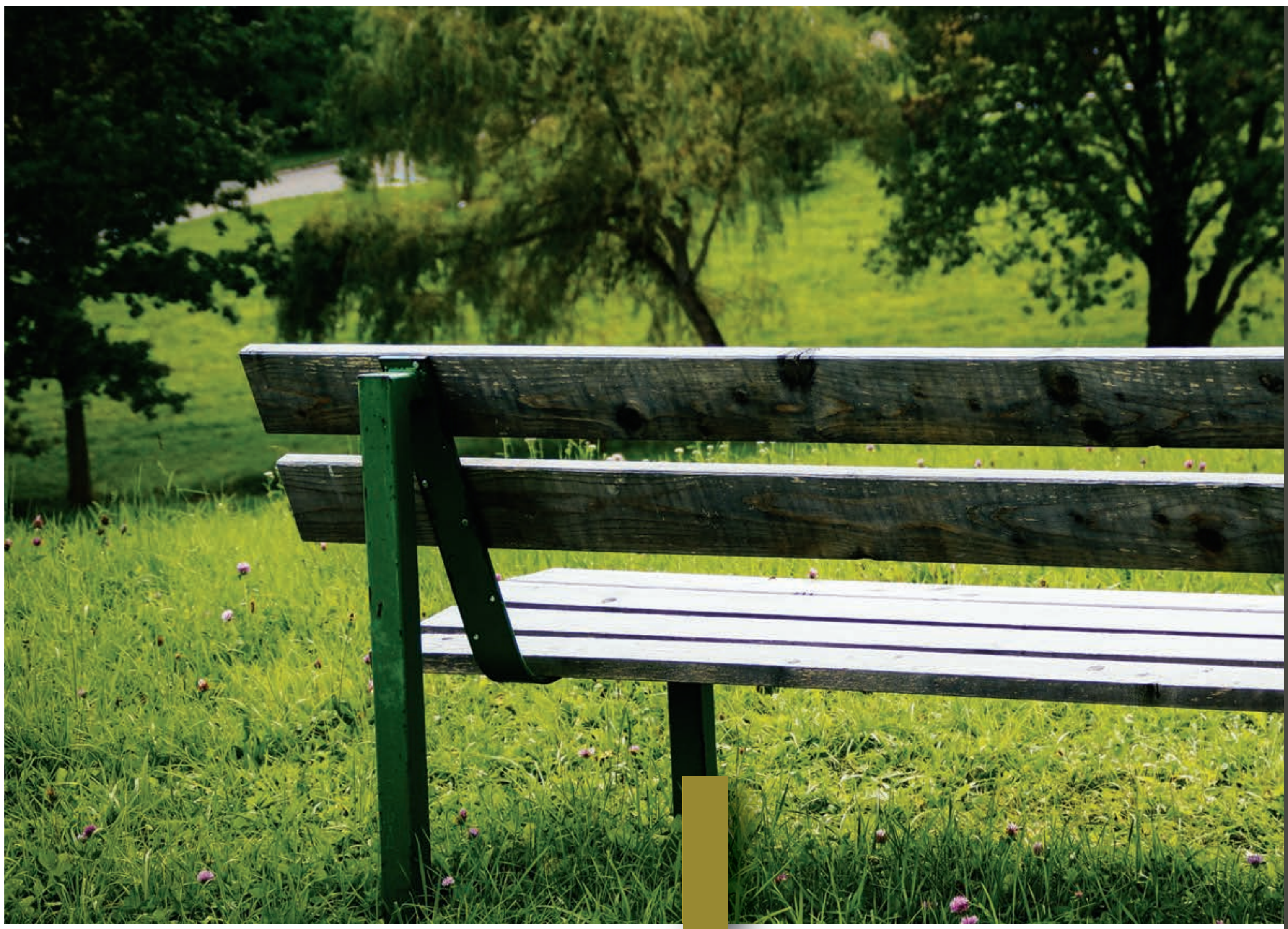

**Descanso**
Venid a mí todos
los que estáis trabajados
y cargados, y yo os haré
descansar.

*(Jesucristo)*

# JULIO 2015

UNIVERSIDAD ADVENTISTA DEL PLATA

/uapargentina @uapargentina

25 de Mayo 99, Libertador San Martín, Entre Ríos. Argentina.
TEL: +54 343 491 8000 - www.uap.edu.ar

| DOMINGO | LUNES | MARTES | MIÉRCOLES | JUEVES | VIERNES | SÁBADO |
|---|---|---|---|---|---|---|
| Notas: | | | **01** El descanso es esencial para gozar de una buena salud. Aprende a disfrutarlo. | ○ **02** El sueño es un fenómeno cíclico natural del cuerpo controlado por el cerebro. | **03** Una persona de 75 años habrá dormido 25 años. ¿No es increíble? | **04** ¿Sabes que el sueño es un aspecto tan esencial para mantener tu salud como lo son comer o respirar? |
| **05** Los niños necesitan dormir entre 9 y 12 horas o más (según la edad), y los adultos, 7-8 horas por noche. ¿Cuánto duermes? | **06** Dormir menos de 6,5 y más de 9 hs por día predisponen a enfermedades cardíacas. En el equilibrio está la salud. | **07** Como un reloj que despierta, el cerebro "avisa" cuándo uno necesita descansar. Respeta los límites de tu cuerpo. | ◑ **08** El sueño adecuado puede ser un factor protector contra la diabetes y dolencias cardiovasculares. | **09** La temperatura corporal, frecuencia cardíaca, producción de hormonas y estado de ánimo son influenciados por el sueño. | **10** Durante el sueño, hay regeneración de células. Duerme bien para rejuvenecer y fortalecer tus defensas. | **11** Aprendes mientras duermes porque dormir bien activa la memoria y la concentración. |
| **12** El rendimiento en el trabajo es afectado por la falta de sueño. Dormir bien previene accidentes. | **13** ¿Sabías que un 60% de los accidentes de tránsito pueden ser prevenidos con una buena noche de sueño? | **14** Una habitación ventilada, tranquila, sin ruidos o humedad y oscura es ideal para un buen dormir. | **15** Las sábanas limpias proporcionan relajación durante el sueño. Lo ideal es lavarlas semanalmente. | ● **16** Si duermes mejor en otro lugar que en tu cama, ¡es la hora de cambiar tu colchón! | **17** Las almohadas, la ropa de cama y el mismo colchón deben recibir sol semanalmente. | **18** Utiliza ropa cómoda para dormir. Descansa de la forma más agradable posible. |
| **19** Televisor, grabadora, computadora y otros equipos en la habitación de descanso comprometen tu sueño. | **20** Para un sueño tranquilo y sin perturbaciones evita mirar películas antes de acostarte a dormir. | **21** Para dormir bien, cena temprano, liviano y sin exageración, por lo menos 2 horas antes de acostarte. | **22** Café, té negro, mate y gaseosas a base de cola son estimulantes y pueden perjudicar el sueño. | **23** El ejercicio físico regular puede promover mejor sueño, ayudándote a dormir rápida y profundamente. | ◐ **24** Una ducha tibia y olor agradable relajan el cuerpo y te hacen descansar mejor. Te sugiero la fragancia de lavanda. | **25** Dormir en horarios regulares, todos los días de la semana, traerá más calidad a tu sueño. |
| **26** Conversación agradable, escuchar música suave o hacer lecturas sanas favorecen el buen sueño. | **27** Treinta minutos de siesta son suficientes para reponer las energías. Esto te ayudará a conciliar el sueño por la noche. | **28** Los fumadores suelen ser candidatos a tener problemas de sueño. Aléjate de este mal. | **29** El alcohol, a la vez que induce el sueño, provoca insomnio durante la madrugada y afecta la capacidad de restauración. | **30** Separa algunos momentos del día para descansar y un día a la semana para recrearte. | ○ **31** Conociste muchos beneficios del descanso adecuado. Aplícalos en tu vida y gana salud y alegría. ¡Dulces sueños! | |

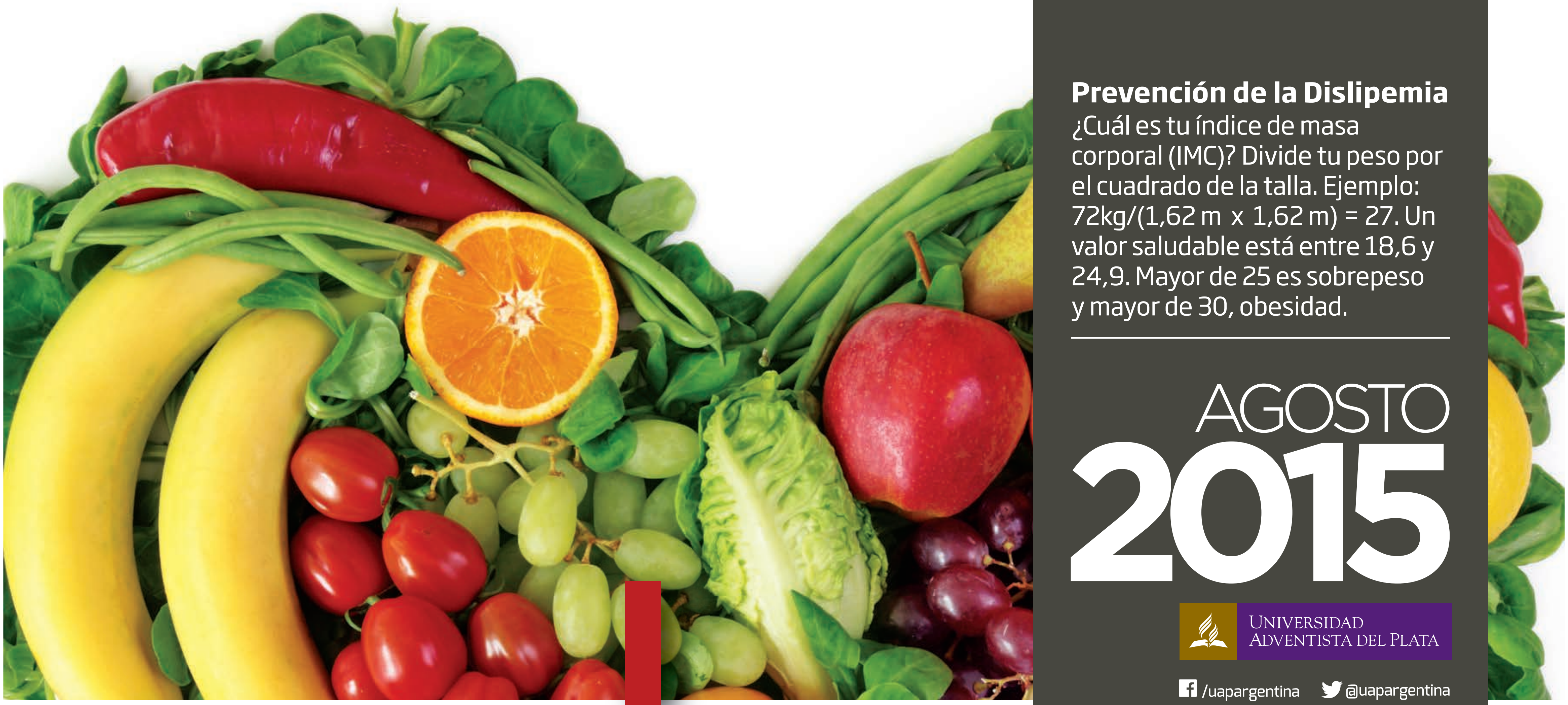

**Prevención de la Dislipemia**
¿Cuál es tu índice de masa corporal (IMC)? Divide tu peso por el cuadrado de la talla. Ejemplo: 72kg/(1,62 m x 1,62 m) = 27. Un valor saludable está entre 18,6 y 24,9. Mayor de 25 es sobrepeso y mayor de 30, obesidad.

# AGOSTO 2015


UNIVERSIDAD ADVENTISTA DEL PLATA

/uapargentina @uapargentina

25 de Mayo 99, Libertador San Martín, Entre Ríos. Argentina.
TEL: +54 343 491 8000 - www.uap.edu.ar


| DOMINGO | LUNES | MARTES | MIÉRCOLES | JUEVES | VIERNES | SÁBADO |
|---|---|---|---|---|---|---|
| **02** Solo los lactantes necesitan de una fuente dietética de colesterol, proporcionada por la leche materna. | **03** Ya has escuchado hablar de las grasas saturadas y las grasas trans, ¿verdad? Normalmente son de origen animal o de grasas procesadas. | **04** Las grasas insaturadas provienen de los vegetales y se relacionan con la prevención de problemas de corazón. | **05** Básicamente, podríamos decir que hay dos clases de colesterol: el bueno (HDL) y el malo (LDL). | **06** Mientras el colesterol HDL protege al corazón y a las arterias, el LDL los daña. | **07** ¡Ojo con la harina blanca y el azúcar en exceso porque aumentan los triglicéridos y la grasa abdominal! | **01/08** El colesterol es esencial para la vida, pero dispensable en la dieta. |
| **09** El ejercicio físico y la dieta sana aumentan el colesterol bueno. ¿Tienes un plan de ejercicios y dieta equilibrada? | **10** Las comidas "chatarra" aumentan el colesterol malo. Mejor evitarlas. | **11** La grasa en exceso es una importante causa de infartos de corazón y de cerebro. Aléjate de este mal. | **12** Consumir grasas saturadas y grasas trans, aunque en pequeñas cantidades, eleva el LDL. Analiza lo que comes. | **13** Mantener el peso ideal te ayuda bajar los niveles de colesterol total, controlar la tensión arterial y la diabetes. | **14** El estar delgado NO es sinónimo de tener colesterol normal. Por ello, hazte un chequeo cada año. | **15** Te desafío a disminuir el colesterol malo y a aumentar el bueno. Te daré algunos consejos. |
| **16** El estrés favorece la producción de sustancias que obstruyen las arterias. Es importante considerarlo. | **17** Un cambio de rutina te ayudará a controlar el estrés. ¿Qué tal recrearse con la familia o amigos? | **18** ¿Fumas? El cigarrillo quita antioxidantes de la sangre y aumenta el colesterol malo y la presión arterial. | **19** Las carnes rojas elevan el colesterol total. Prefiere las carnes magras. | **20** Las frutas, verduras, legumbres y cereales integrales ayudan a controlar el colesterol. ¿Ya los consumiste hoy? | **21** Las semillas de chía, lino y las nueces, ricas en Omega 3, previenen problemas cardiovasculares. ¿Ya las probaste? | **22** Los fitoesteroles, encontrados en los vegetales, reducen el LDL. Reserva la mitad de tu plato para ellos. |
| **23** ¡Ojo con la crema de leche, quesos, yema de huevo, hígado y piel de pollo! Son comidas ricas en LDL. | **24** Los problemas cardíacos son más frecuentes en quienes comen carnes, quesos y lácteos enteros. | **25** Recuerda que el ejercicio, además de ayudarte a controlar el colesterol y los triglicéridos, te deja en forma. ¡A ejercitarse! | **26** Camina a diario y consume fibras naturales. Persistir es clave para formar hábitos saludables. ¡Adelante! | **27** Continúa tomando tus 8 vasos de agua diarios. Recuerda tomar uno de ellos en ayunas. | **28** Agrega frutas y verduras crudas a tu dieta. Tu salud agradecerá. | **29** Parte del colesterol es desechado con las heces. Idealmente, deberíamos ir de cuerpo al menos una vez al día. |
| **30** ¿Sigues con tu plan de caminatas? El ejercicio te aumenta el HDL. No desistas. | **31** Repasa nuevamente cada consejo para incorporarlos a tu diario vivir. ¡Adelante, vas a tener mejor salud! | Notas: | | | | |

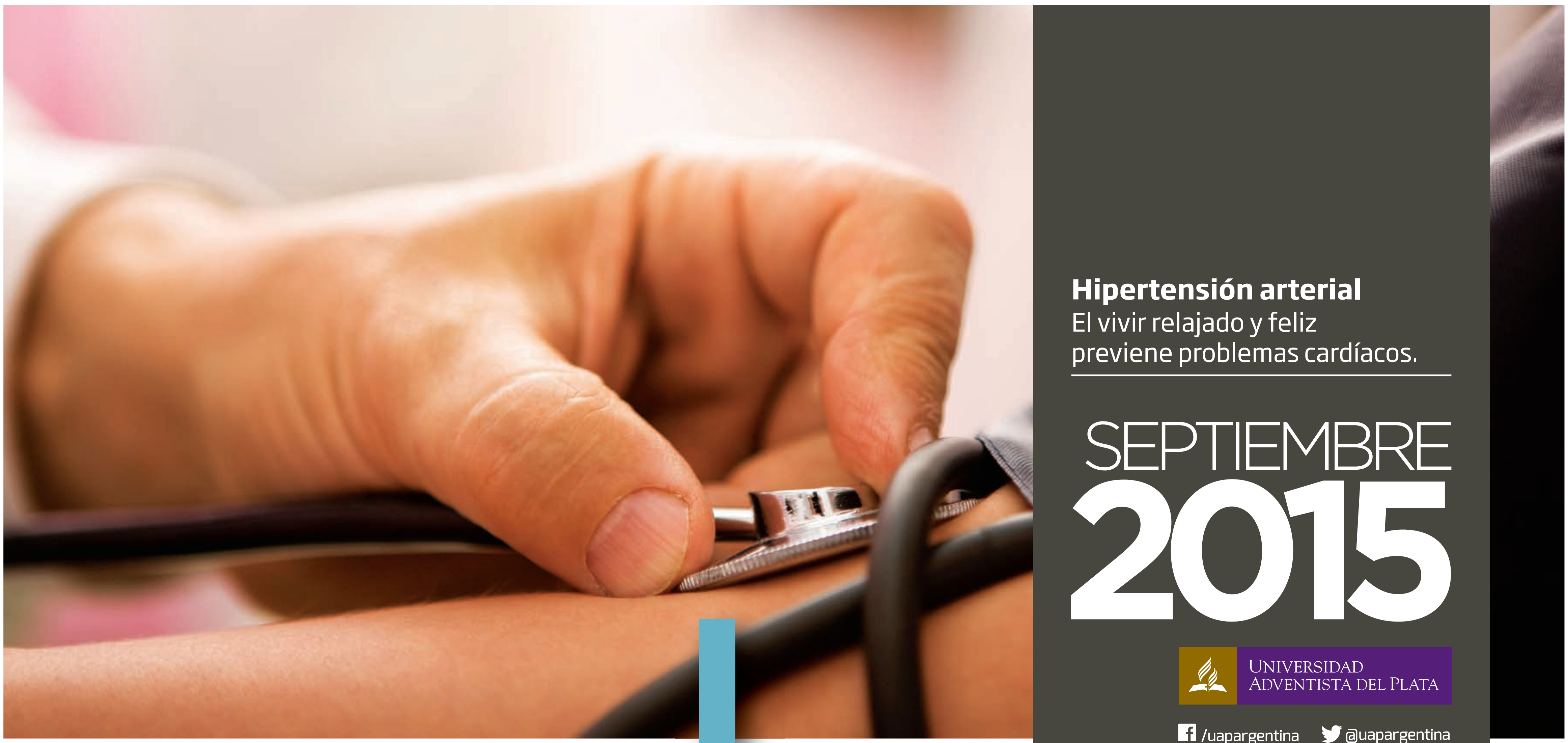

**Hipertensión arterial**
El vivir relajado y feliz
previene problemas cardíacos.

# SEPTIEMBRE 2015

UNIVERSIDAD ADVENTISTA DEL PLATA

/uapargentina @uapargentina

25 de Mayo 99, Libertador San Martín, Entre Ríos. Argentina.
TEL: +54 343 491 8000 - www.uap.edu.ar

| DOMINGO | LUNES | MARTES | MIÉRCOLES | JUEVES | VIERNES | SÁBADO |
|---|---|---|---|---|---|---|
| | **Unidad de tensión arterial | **01** La hipertensión arterial es la segunda causa de muerte en la Argentina. ¡Préstale mucha atención a tu presión! | **02** La hipertensión es un "asesino silencioso" porque a menudo no da síntomas, no se la percibe elevada. | **03** Se dice que hay hipertensión cuando la presión es igual o superior a 140/ 90 mm Hg** (milímetros de mercurio) en 3 ocasiones distintas. | **04** La presión controlada disminuye el riesgo de enfermedades cardiovasculares. ¿Cómo anda tu presión? | **05** Controla tu presión con frecuencia. |
| **06** La risa hace bajar la presión, combate el estrés y favorece la digestión. | **07** El exceso de sal, grasa, tabaquismo, obesidad, sedentarismo, diabetes y estrés se relacionan con la hipertensión. | **08** Un adulto sano puede consumir hasta un máximo de 5g diarios de sal mientras a un niño menor de 1 año no se le debe dar sal. | **09** Una buena noticia: la hipertensión puede ser prevenida y tratada. | **10** La clave de la prevención y del tratamiento de la hipertensión es atacar las causas conocidas. | **11** La sal eleva la presión. El paladar se acostumbra si usas poca sal en las comidas. ¡Inténtalo! | **12** Prueba condimentar tus comidas con hierbas aromáticas y limón. Con esto podrás reducir la sal sin comprometer el sabor. |
| **13** Los alimentos en conserva y los ahumados poseen mucha sal. ¡Evítalos! Los frescos son más saludables. | **14** La grasa animal tapa las arterias y eleva la presión. Prefiere carnes magras y aceites vegetales no fritos. | **15** Cuida con los alimentos enlatados, porque son muy ricos en sal. Prefiere los frescos, siempre. | **16** Las fibras encontradas en las frutas, verduras, legumbres y cereales benefician la presión. | **17** Algunos controladores de la presión: ajo, lechuga, brócolis, vegetales rojos y anaranjados, soja, salvado de trigo y avena. | **18** El tabaco es el principal factor de riesgo para enfermedades cardíacas. Dejar de fumar mejora la presión. | **19** Lo bueno es que cuando dejas de fumar la presión tiende a normalizarse. ¿Por qué no "apagas" este vicio? |
| **20** Un peso saludable ayuda a mantener la presión arterial dentro de rangos normales. | **21** Controla tu azúcar en la sangre para mantener buena la presión. | **22** Acuérdate que la obesidad es un factor que facilita la diabetes y la hipertensión. Busca ayuda para bajar de peso. | **23** Estar físicamente activo es buena medida para disminuir la presión y el peso. La caminata es el ejercicio por excelencia. | **24** El ejercicio baja el azúcar en la sangre y el colesterol. ¿Tienes un plan de ejercicio? | **25** Te desafío a caminar 30 min. diarios por lo menos 3 veces en la semana. ¡Muévete! | **26** El estrés es otro desencadenante de presión alta. Enfrenta con optimismo tus desafíos. |
| **27** Prefiere los alimentos horneados a los fritos. Tu corazón te lo agradecerá. | **28** El alcohol, café, té y refresco de cola pueden alterar la presión arterial. Disminuye el consumo de estos estimulantes. | **29** Si eres hipertenso, ojo con el agua que tomas. Puede tener mucho sodio, que aumenta tu presión arterial. | **30** Ah!, y no te olvides de cambiar los productos lácteos enteros por los descremados. | Notas: | | |

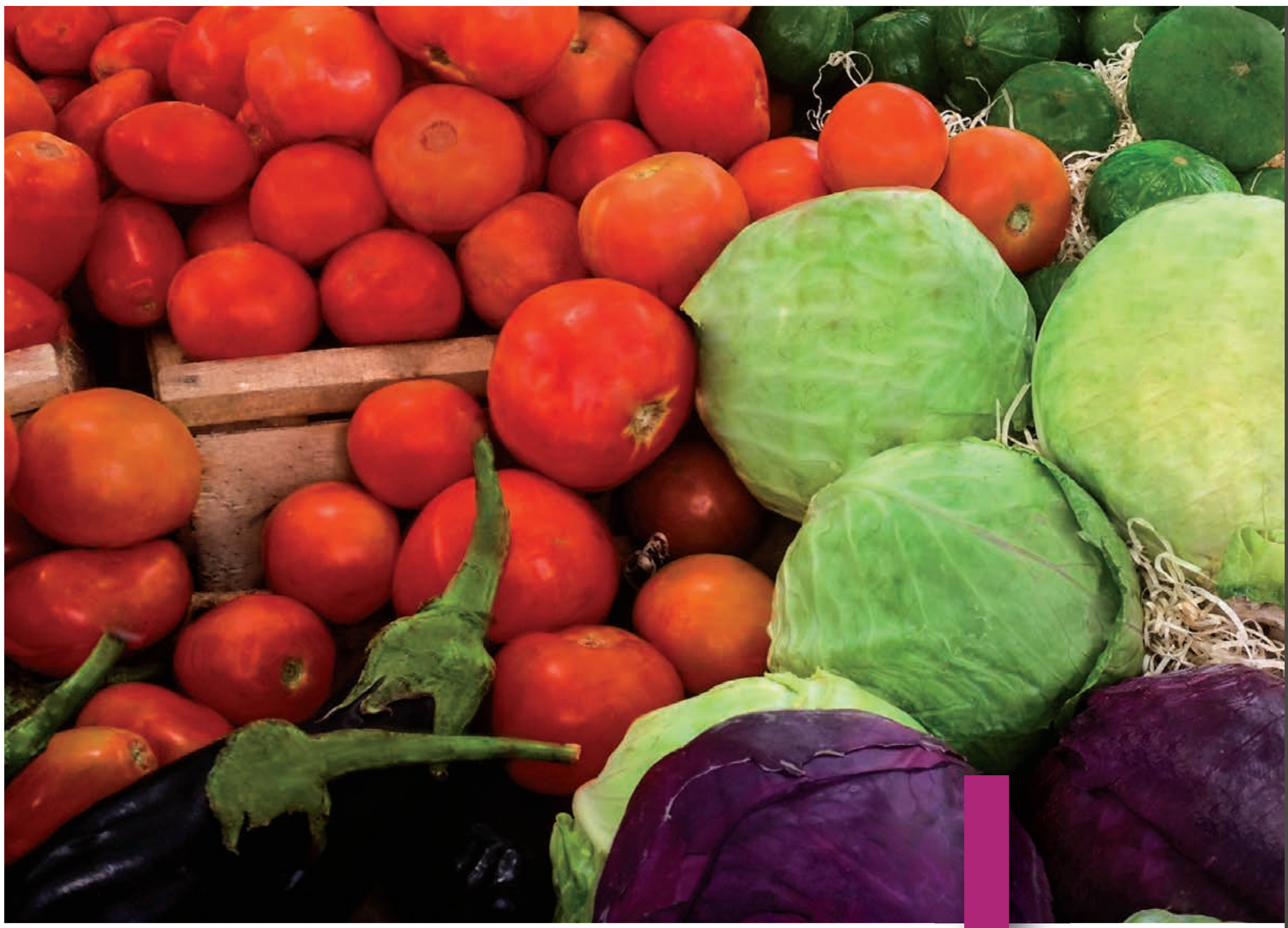

**Diabetes**

Los genes puede que carguen la pistola, pero es la conducta humana la que aprieta el gatillo.

*(Frank Vinicor)*

# OCTUBRE 2015


Universidad Adventista del Plata

/uapargentina @uapargentina

25 de Mayo 99, Libertador San Martín, Entre Ríos. Argentina.
TEL: +54 343 491 8000 - www.uap.edu.ar


| DOMINGO | LUNES | MARTES | MIÉRCOLES | JUEVES | VIERNES | SÁBADO |
|---|---|---|---|---|---|---|
| Notas: | | | | **01** Desafortunadamente la diabetes se ha convertido en una enfermedad muy común. | **02** La buena noticia es que además de ser tratable, la diabetes muchas veces es evitable. | **03** El diabético tiene niveles altos de azúcar en sangre (glucemia). ¿La has controlado? |
| **04** El valor normal de azúcar en la sangre (glucemia), en ayunas, es de 70 a 110 mg/dl. | **05** Sed, hambre y orina excesivas pueden ser señales de diabetes. Busca tu médico si las detectas. | **06** La diabetes puede afectar a muchos órganos como el cerebro, el corazón, el riñón y los ojos. | **07** ¿Quieres prevenir la diabetes? Lo primero para hacer es revisar tu dieta. | **08** Las comidas ricas en azúcares, gaseosas y harinas refinadas aumentan rápidamente los niveles de azúcar en la sangre. | **09** Las carnes rojas y grasas tienen fuerte relación con la diabetes. Los vegetales crudos o poco procesados la previenen. | **10** Dietas a base de porotos (judías o frijoles), chauchas y otras legumbres, reducen el riesgo de padecer diabetes. |
| **11** Maní, girasol, maíz, aceituna, nueces y otras oleaginosas, favorecen niveles de azúcar más bajos en la sangre. | **12** Comer 2 a 3 frutas chicas por día, con cáscara, es un buen aporte de fibras. | **13** Si eres diabético, limita el consumo de banana, uva e higo ya que son ricas en azúcar. | **14** Los alimentos sin agregados de azúcar pueden tener un alto contenido de carbohidratos. ¡Controla la etiqueta! | **15** El ejercicio físico es excelente para controlar la glucemia. ¿Qué tal empezar con una caminata de 20 minutos? | **16** El ejercicio físico previene y trata la diabetes. ¡Sorprendente! Una razón más para moverse. | **17** Si eres diabético o conoces a alguien que la padece, ten en cuenta los consejos a continuación. |
| **18** Inspecciona los pies antes del ejercicio y después de él. Ten cuidado para no lastimarlos. | **19** Usa zapatillas y ropas confortables. ¿Qué bueno es estar cómodo! | **20** Usa dos pares de medias de algodón o un par bien acolchonado y absorbente, con las costuras hacia afuera. | **21** Toma agua antes del ejercicio, durante y después de él. | **22** El diabético normalmente orina abundantemente. Mantente hidratado. Que la botellita con agua te acompañe siempre. | **23** El sobrepeso y la obesidad son factores de riesgo para desarrollar diabetes. ¿Está tu peso acorde con tu talla? | **24** ¿Andas muy estresado? El estrés aumenta el azúcar en la sangre. ¿Puedes encontrar motivos para reírte hoy? |
| **25** Afrontar los desafíos con confianza en Dios, calma la ansiedad y el estrés, y ayuda a controlar la glucemia. | **26** Los adultos que duermen de 7 a 8 horas por noche tienen menor riesgo de desarrollar diabetes. | **27** La luz solar estimula la producción de vitamina D que disminuye la glucemia. ¿Qué tal un baño de sol? | **28** El diabético puede llevar una vida saludable y normal. Busca la orientación de tu médico. | **29** Recuerda que una dieta equilibrada y actividad física ayudan a tratar y a prevenir la diabetes. | **30** Si eres diabético deberás mantener tu presión arterial por debajo de 135/80, para el bien de tu corazón, riñones y ojos. | **31** Visita a tu médico anualmente y hazte un chequeo. Controla tu glucemia. |

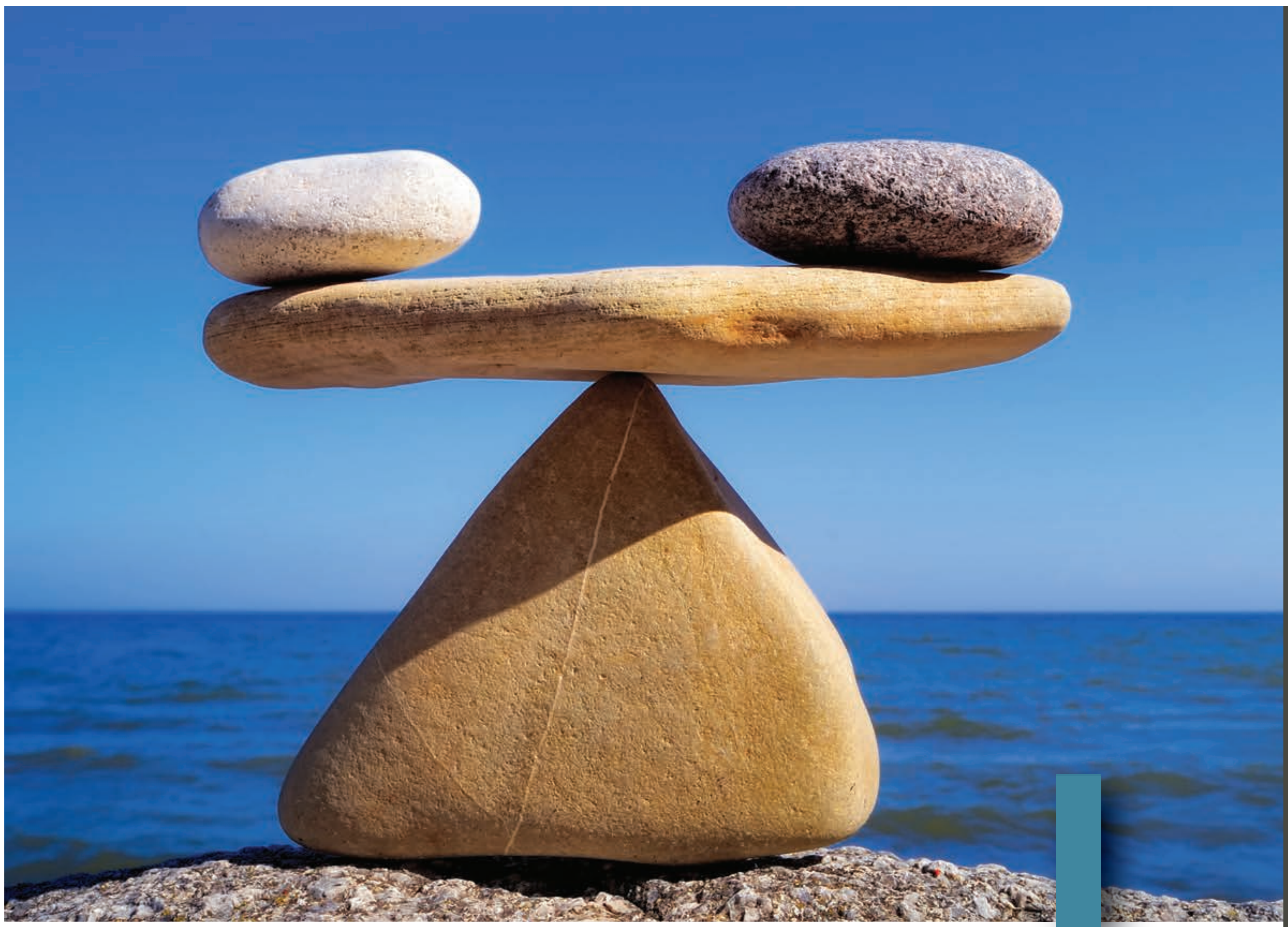

**Temperancia**
Hay una fuerza motriz más poderosa que el vapor, la electricidad y la energía atómica: la voluntad.
*(Albert Einstein)*

# NOVIEMBRE 2015

| DOMINGO | LUNES | MARTES | MIÉRCOLES | JUEVES | VIERNES | SÁBADO |
|---|---|---|---|---|---|---|
| **01** La clave para añadir vida a tus años es hacer sabio uso de las cosas buenas y dejar de lado todo lo perjudicial para la salud. | **02** ¡El cigarrillo va matar 1 billón de personas en este siglo! Pero tú puedes estar fuera de estos números. | **03** ¿Sabías que por cada cigarrillo fumado se restan 11 minutos de vida? | **04** Con 20 minutos sin fumar la presión tiende a normalizarse. ¡Prepárate para gozar los muchos beneficios de no fumar! | **05** Con 20 minutos sin fumar la presión tiende a normalizarse. ¡Prepárate para gozar los muchos beneficios de no fumar! | **06** El tabaco predispone al cáncer de laringe y es más dañino a la piel que el exceso de sol. | **07** Los problemas de corazón y pulmón son las principales causas de muerte en fumadores. Pero es posible vencer este vicio. |
| **08** Mañana escribirás una nueva página en tu historia. ¡Tu salud, autoestima y bolsillo se beneficiarán por no fumar! | **09** Hoy es el ¡gran día! Afirma para ti mismo: HOY HE DECIDIDO DEJAR DE FUMAR. | **10** Si no fumas, ¡felicitaciones! Gana salud ayudando a alguien a dejar de fumar. | **11** Tomar agua, ejercitarse, comer sano y evitar café y alcohol te ayudará a dejar de fumar. | **12** ¡Guau! 48 horas sin fumar te disminuyeron el riesgo de muerte súbita. Increíble. ¿Qué tal un día más sin fumar? | **13** Hoy puede ser el día más crítico y aunque tu cuerpo "suplique" por el cigarrillo, puedes vencer. | **14** Ocho vasos de agua, ejercicios y una ducha tibia antes de acostarse, te ayudarán a relajar para proseguir. |
| **15** ¡Buen día! ¿Hueles algo distinto? Tu gusto también ha mejorado. Saborea tu fruta preferida. | **16** ¿Viste?, es posible. Sigue un día más sin humo. Repite tu decisión y evita todo lo que te recuerda al cigarrillo. | **17** ¡Eres un victorioso! Hace 7 días que aceptaste el desafío. Si llegaste a fumar, no te desanimes. ¡Hoy puedes vencer! | **18** Continúa gozando de la alegría de ser el dueño de tu voluntad. Permanece fiel a tu decisión. | **19** Tus queridos te apoyan. Cuenta a la gente que dejaste de fumar. ¡Que tengas un exitoso día! | **20** ¡Gran victoria! Para continuar exitoso/a, recuerda: evita los estimulantes (café, alcohol, picantes, sal, etc.). | **21** Prefiere los alimentos naturales, toma mucha agua, sigue con las caminatas y proclama que ¡eres libre del vicio! |
| **22** Sigue firme en tu plan. No te preocupes por el mañana. Vive hoy feliz con tu decisión. | **23** Si no lograste tu propósito, tranquilo: puedes empezar hoy nuevamente. Vive un día a la vez. | **24** Hay una sustancia relacionada con violencia y destrucción de familias: el alcohol, incluso el del vino. | **25** El resveratrol presente en la uva, es un potente antioxidante que sí protege al corazón. | **26** El jugo de uva aporta todo lo bueno del vino sin los perjuicios del alcohol. ¿Qué te parece? | **27** No hay cantidad segura. Aún en pequeñas cantidades, el alcohol destruye partes significativas del organismo. | **28** En mujeres, pequeñas dosis diarias de alcohol pueden producirles cirrosis del hígado y aumentan el riesgo de aborto espontáneo. |
| **29** Sé el dueño de tus decisiones, asume el control de tus actitudes. Si el vicio te está ganando, busca ayuda de un profesional calificado. | **30** Escucha a tu familia. Ellos pueden ser una contención en el momento de las dificultades. | | | | | |

Notas: ______________________

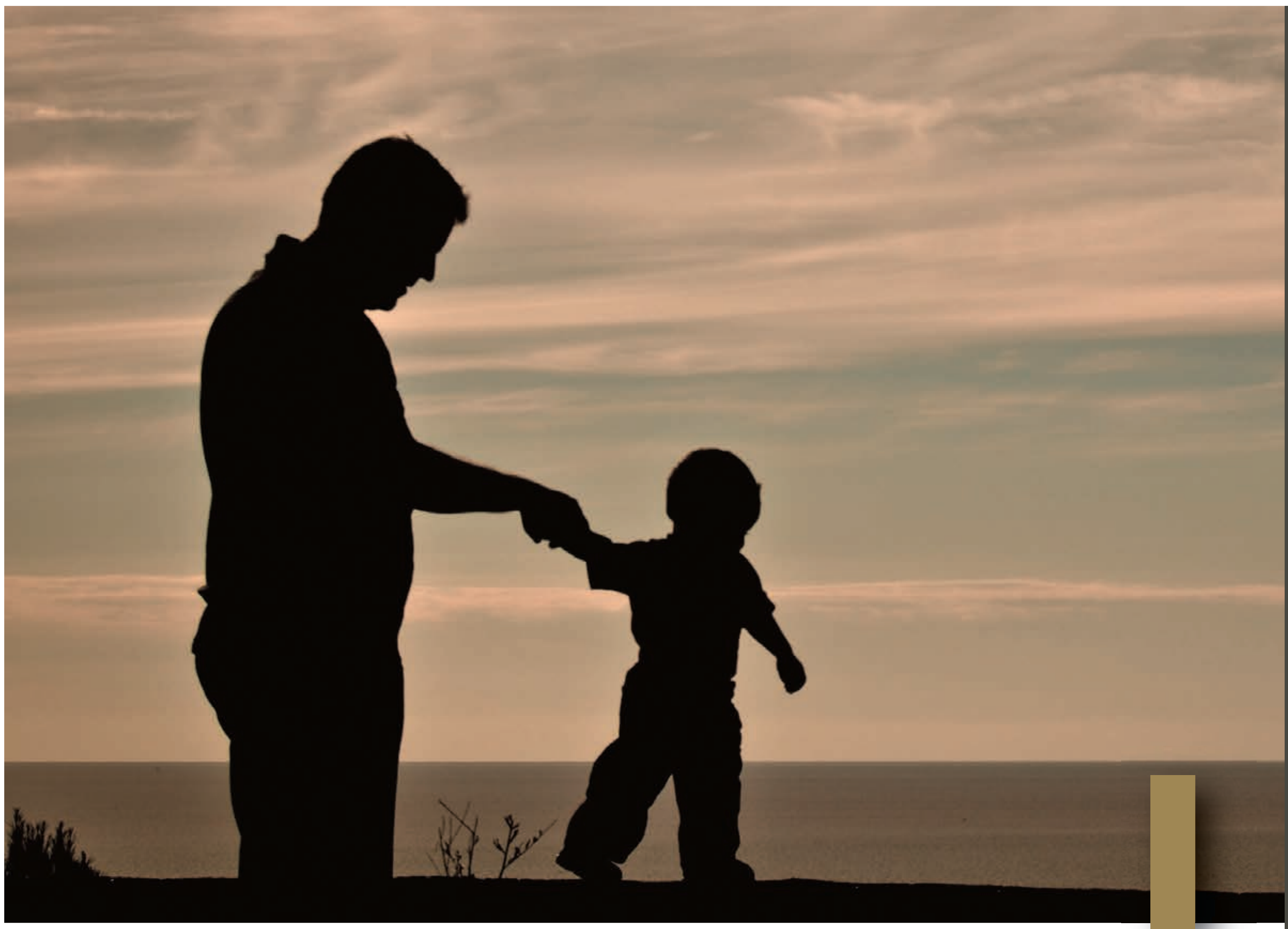

**Esperanza**
Si supiera que el mundo
se habría de acabar mañana,
yo hoy todavía plantaría
un árbol.
*(Martin Luther King)*

# DICIEMBRE 2015

Universidad Adventista del Plata

/uapargentina @uapargentina

25 de Mayo 99, Libertador San Martín, Entre Ríos. Argentina.
TEL: +54 343 491 8000 - www.uap.edu.ar

| DOMINGO | LUNES | MARTES | MIÉRCOLES | JUEVES | VIERNES | SÁBADO |
|---|---|---|---|---|---|---|
| **Notas:** | | **01** Amigo, te animo a conocer y aplicar otra dimensión a tu estilo de vida: la esperanza. | **02** A pesar de los problemas es posible encarar la vida con optimismo. | **03** Lo primero a que te voy a invitar es que disfrutes del mejor día que hay para vivir: ¡HOY! | **04** Hay una conexión vital entre cuerpo y mente. ¡Somos aquello que pensamos! | **05** Muchas enfermedades son producidas por pensamientos enfermizos. ¿Con qué nutres tu mente? |
| **06** ¡Mucha gente que vive enferma sería sana si tan solo así lo creyera! | **07** Valorar la familia y vivir en amor son una salvaguarda contra la desesperación. | **08** ¿Sabías que adultos que se relacionaron bien con sus padres desarrollan menos enfermedades? | **09** Las personas que tienen placer en la familia poseen menor probabilidad de desarrollar Alzheimer. | **10** ¿Tienes sueños? Quienes luchan por alcanzarlos presentan mejores defensas contra el cáncer. | **11** No importa la edad que tengas. Haz proyectos y empéñate a realizarlos. Tu vida cobrará más sentido. | **12** No tener problemas no necesariamente significa ser feliz. Las personas sin desafíos casi siempre son infelices. |
| **13** Servir al prójimo mejora la salud física y mental, y la esperanza de vida. | **14** Mientras más se demuestra el amor en la vida práctica más se disminuyen los niveles de estrés. | **15** Ayudar a los que necesitan mejora la autoestima, la autoconfianza y añade fe al futuro. | **16** ¿Quieres tener bienestar? Atrévete a ayudar a los demás. | **17** El altruismo, dedicarse al prójimo, es uno de los factores más significativos para dar sentido a la vida. | **18** ¿Sonreíste hoy? La sonrisa es un excelente cosmético. Alegra la vida de quien la recibe y rejuvenece al que la dona. | **19** Vivir alegre es una medicina por excelencia. Aumenta las defensas y disminuye la ansiedad. |
| **20** ¡Sorprendente! El perdón y la alegría previenen al cáncer. ¿Estás dispuesto a perdonar hoy? | **21** ¿Tienes amigos? Mantener buenas relaciones son vitales para mejorar la salud física y mental. | **22** La amistad no es una gran cosa, sino un millón de pequeñas cosas, dijo alguien. | **23** ¡Un amigo es lo más lindo que se puede tener y una de las mejores cosas que puedes ser! | **24** Pero, ¿cómo hacer amigos? Simple. Involúcrate con la gente y sé el tipo de amigo que deseas que sean los otros. | **25** ¿Le dijiste hoy a alguien que lo/a quieres? Acuérdate que hoy es el día ideal para decirlo. | **26** Mira qué increíble. Personas que sufrieron un ataque del corazón tienen menor riesgo de muerte si se sienten contenidas. |
| **27** Otro factor de cura reconocido por la Medicina es creer en Dios. | **28** Las personas practicantes de una religión mueren menos por cáncer y por enfermedades cardíacas. ¡Interesante! | **29** Las personas que oran y frecuentan una iglesia tienen una expectativa de vida de 7 años más que aquellos que no lo hacen. | **30** La dieta, el estilo de vida sano y la espiritualidad disminuyen el riesgo de accidente cerebrovascular y demencia. | **31** "Si ayudo a una sola persona a tener esperanza, no habré vivido en vano" (M. L. King). ¡Compártelo! | | |

# Datos de identificación del
# Calendario de Salud
# 2105

**Centro de Investigación para Reducir Desigualdades en Salud (CIRDES)**
Facultad de Ciencias de la Salud - Universidad Adventista del Plata
Entre Ríos, Argentina

*Cada día del año de este calendario presenta un mensaje de salud redactado en base a una criteriosa búsqueda bibliográfica en la literatura médico-científica. El principal propósito del calendario es despertar el interés por la salud integral, promoviendo cambios positivos en el estilo de vida. Las informaciones de este calendario no son presentadas para reemplazar la atención médica de salud o para servir de guía para la automedicación. Las recomendaciones del calendario son generales y es posible que no se apliquen a determinadas condiciones y particularidades de salud de algunas personas.*

**Logros:**
Nuestra propuesta es que cada día del año puedas acompañar los mensajes marcando los días en donde encontraste informaciones novedosas sobre salud. Al fin de cada mes, te sugerimos reunir con tu familia y escribir en el espacio de notas mensuales los logros alcanzados con respecto al tema del mes.

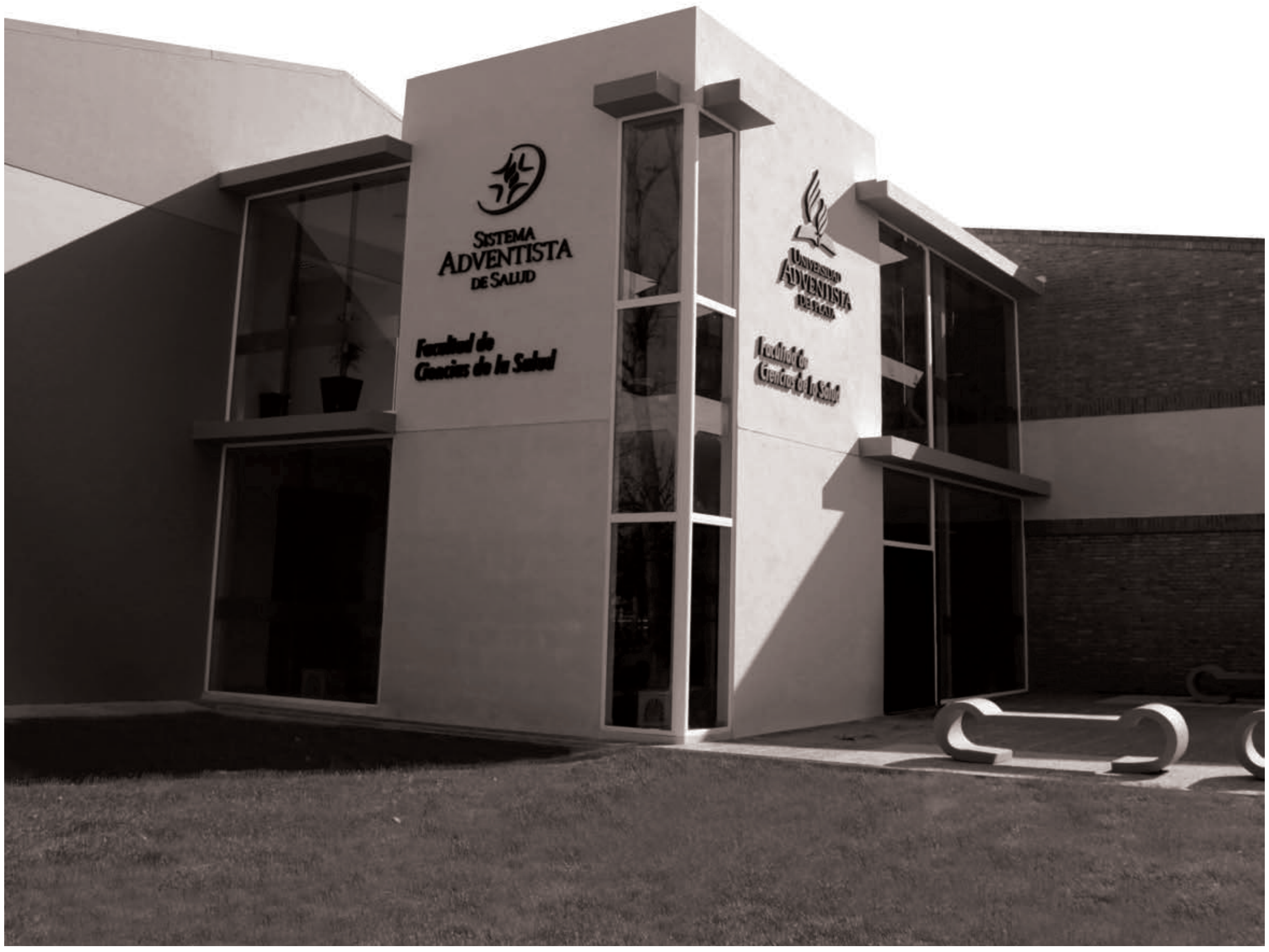


En caso de duda, sugerencias o alguna aclaración, favor contactarnos a través del teléfono **(0343) 491-8000, interno 1236,** o del correo electrónico **cienciaytecnica@uap.edu.ar**
Este calendario es una cortesía de la Universidad Adventista del Plata y no debe ser comercializado.

Universidad Adventista del Plata
